# Novel insights: crosstalk with non-puerperal mastitis and immunity

**DOI:** 10.3389/fimmu.2024.1431681

**Published:** 2024-08-01

**Authors:** Yao Zhou, Jie Gong, Xianguang Deng, Lele Shen, Lifang Liu

**Affiliations:** ^1^ Department of Galactophore, The First Hospital of Hunan University of Chinese Medicine, Changsha, Hunan, China; ^2^ Hunan University of Chinese Medicine, Changsha, Hunan, China

**Keywords:** non-puerperal mastitis, granulomatous lobular mastitis, plasma cell mastitis, immune cell, prolactin, immunity, review

## Abstract

The two primary types of non-puerperal mastitis (NPM) are granulomatous lobular mastitis (GLM) and plasma cell mastitis (PCM). Existing research indicates that immune inflammatory response is considered to be the core of the pathogenesis of GLM and PCM, and both innate and adaptive immune responses play an important role in the pathophysiology of PCM and GLM. However, the regulatory balance between various immune cells in these diseases is still unclear. Consequently, we present a comprehensive summary of the immune-related variables and recent advances in GLM and PCM.

## 1 Introduction

In recent years, there has been a significant increase in the prevalence of Non-Puerperal Mastitis (NPM) (1, 2). However, there is a notable lack of comprehensive and large-scale epidemiological investigations pertaining to NPM, as well as a shortage of clinical and basic research in this field (3). The etiology of NPM is largely unknown, and its high recurrence rate and treatment challenges pose significant difficulties for both clinicians and patients (4).

NPM is characterized as a benign inflammatory disease that occurs in women during the non-lactating period, which mainly includes two distinct conditions: granulomatous lobular mastitis (GLM) and plasma cell mastitis (PCM) (5, 6). Although these two diseases share certain clinical manifestations, such as breast lumps, pain, erythema, abscesses, ulcers, and fever, there are also distinct differences between them (7). GLM typically presents with breast lumps outside the areola. Conversely, PCM often manifests as lumps beneath the areola, which can lead to pus-filled and ulcerated ductal fistulas (8). Furthermore, GLM is more prevalent among women of childbearing age, while PCM can affect women of all ages. Nevertheless, a pathological biopsy of the breast mass remains the gold standard for distinguishing between these two diseases.

PCM is also known as ductal dilatation and periductal mastitis, depending on the course of the disease (9). The characteristic histopathologic manifestations of PCM are infiltration of large numbers of plasma cells with dilatation of the mammary ducts, and inflammatory changes in the ductal walls and periductal tissues (5). GLM also referred to as idiopathic granulomatous mastitis (IGM), is characterized by inflammatory granulomas centered on the lobules of the mammary glands with infiltration of lymphocytes, plasma cells, and neutrophils (10, 11).

T-lymphocytes, which express the surface protein CD3, are integral components of the immune system (12). Several studies have utilized immunohistochemical staining with CD3 antibodies to observe localized infiltration of CD3-positive T-lymphocytes in GLM breast mass tissues (6, 13, 14). This finding strongly supports the correlation between GLM and localized adaptive immune responses in the breast. Another study confirmed the presence of macrophage activation in both GLM and PCM biopsy specimens (15), highlighting its significance in the intrinsic immune response. Additionally, impaired ductal secretions excretion and hyperprolactinemia may trigger localized immune-inflammatory responses in the mammary gland, contributing to the development of GLM or PCM (16–18). Therefore, the localized immune-inflammatory responses within the lesions may play a crucial role in the pathogenesis of GLM and PCM.

Based on the above background, we reviewed the roles of relevant cells and molecules in the adaptive and intrinsic immune systems in NPM, respectively, and delved into the crosstalk between NPM and other immune factors, such as elevated prolactin, local hypersensitivity due to milk retention, lipid metabolism disorders, and immune-inflammation-related pathways.

## 2 Immune cells in normal mammary glands

The normal mammary gland is composed of 15–20 glandular lobes, which are separated by connective tissue and converge through milk ducts. The end of the glandular lobe is divided into many mammary lobules, which are connected to the acini by lobular ducts (19). While lactating, the acini can secrete milk. Testing the normal breast tissue surrounding breast cancer and breast samples following preventive resection, Ruffell et al. discovered that the normal breast tissue had an immune microenvironment dominated by T lymphocytes (20); Recently, a single-cell sequencing research has thoroughly examined the cells in normal breast tissue (21). The findings indicate that perivascular cells, endothelial cells, monocytes/macrophages, T and B lymphocytes, and fibroblasts make up the normal mammary ductal microenvironment. These immune cells can contribute to chronic inflammation and immunological surveillance. They are also capable of recognizing novel antigens and both endogenous and exogenous ligands.

**Figure 1:**
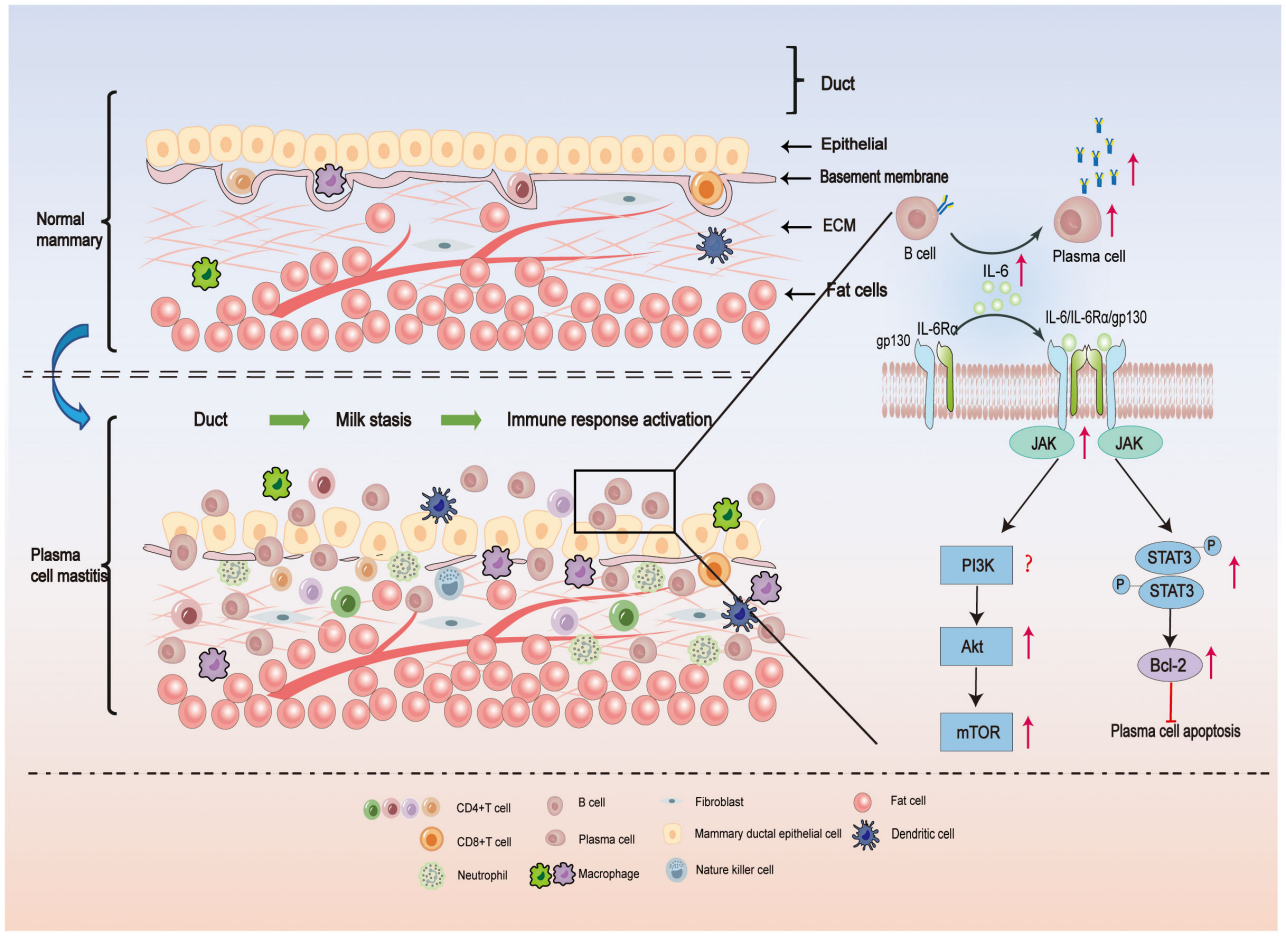
In the upper part of **Figure 1**, the local microenvironment of a normal mammary duct is shown schematically. The epithelial cells of the normal ducts are tightly packed, the epithelial basement membrane is continuous, and a very small number of immune cells are present locally in the tissue. In the lower part of **Figure 1**, the local microenvironment of the ducts of PCM is illustrated. Milk stasis exists in the ducts of PCM, and lipids and antibodies in the milk can activate the local immune response, resulting in damage to the epithelial cells of the ducts, enlarged cell gaps, and incomplete epithelial basement membranes, with a large number of plasma-cell-based inflammatory cells infiltrating the tissues. IL-6 plays an important role in PCM by promoting the differentiation of B cells into plasma cells and the release of antibodies. In addition, when IL-6 binds to IL-6R on the cell surface, Janus kinase (JAK) is phosphorylated, and then STAT3 is phosphorylated, which promotes downstream Bcl-2 expression and inhibits plasma cell apoptosis. On the other hand, activated JAK activates the PI3K/Akt/mTOR signal pathway, which induces an inflammatory response and is involved in the pathogenesis of PCM (23, 24).

It’s intriguing because Degnim AC et al. used immunohistochemical labeling to investigate the location of immune cells in normal breast tissue as early as 2014 (22). They discovered that in normal breast tissue, dendritic cells (DCs) and cytotoxic T cells are present in the mammary duct epithelial cells, but monocytes, macrophages, and lymphocytes are primarily found in the mammary lobules. However, the ratio of monocytes/macrophages to dendritic cells in breast tissue remained unchanged in breast lobular inflammation, while the number of adaptive immune (T and B) cells increased significantly. This suggests that adaptive immunity may be the primary cause of breast lobular inflammation. (**Figure 1** depicts the normal breast anatomy and the distribution of immune cells within it).

## 3 Roles of adaptive immune cells and molecules in NPM

### 3.1 T helper cells

**Figure 2:**
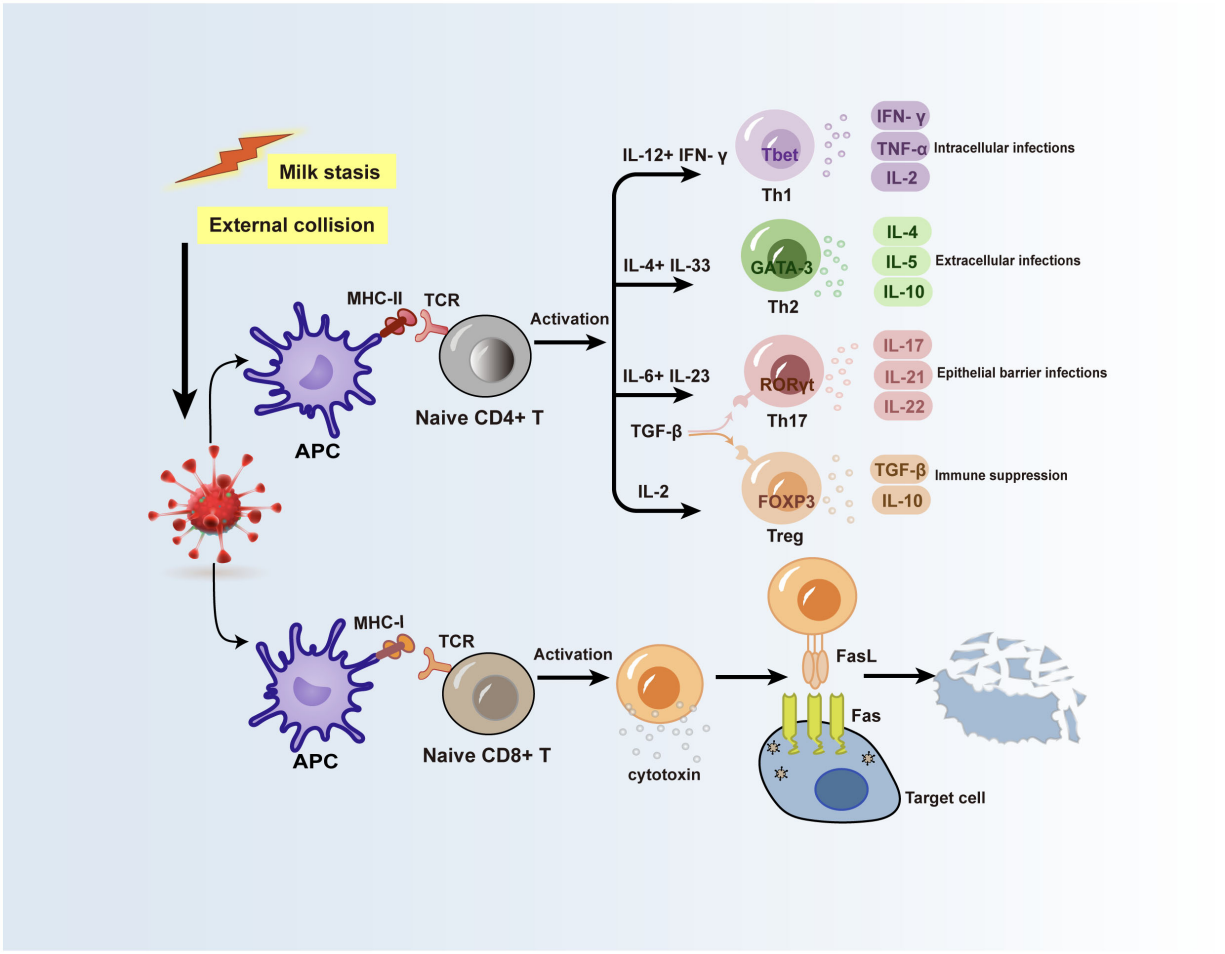
Possible mechanisms for the role of T lymphocytes in GLM and PCM. Inducible factors such as stagnant ductal secretions or external collisions can lead to local mammary epithelial cell damage, and autoantigens in ductal secretions are taken up by APCs and processed into antigenic fragments. T cell receptors on the surface of naive CD4+ T cells and naive CD8+ T cells recognize MHC-II molecules and MHC-I molecules on the surface of APCs, respectively, and acquire antigenic fragments, while the activation of these two types of cells produces various immune cytokines or molecules. Inflammatory factors produced by Th cell subsets subsequently activate cellular or humoral immunity, and CD8+ T cells act on target cells infected with antigens through the surface FasL receptor to cause target cells to lyse. APC, antigen-presenting cell; TCR, T cell receptor; FasL, Fas ligand.

T helper cells (Th cells), also known as CD4+ T-cells, are crucial parts of the adaptive immune system (25). Upon the entry of foreign antigens into the body, antigen-presenting cells (APCs) sequester and transform them into antigenic fragments. Subsequently, the T-cell receptors on the Th cells’ membrane recognize the major histocompatibility complex-II (MHC-II) molecules on the APCs’ surface and acquire the antigenic fragment, initiating the immune system’s response (26). At the same time, the Th cells are activated to produce typical cytokines (27), and unique Th cell subsets can be identified by searching for specific priming cytokines, major transcription factors, and distinctive cytokines (28, 29) (**Figure 2**). To efficiently aid in antigen clearance, Th cell subpopulations modify their cytokine function library following their homologous antigens. However, polarized Th cell cytokine patterns might facilitate tissue damage and pathology in the event of immunological rejection, resulting in allergies or autoimmune reactions. Researches currently show that the serum microenvironment of GLM patients contains a variety of T cell subsets (30–32). According to more studies, certain cytokines released by Th1, Th2, and Th17 cell subsets may be crucial in the development of PCM and GLM (**Figure 2**).

#### 3.1.1 Th1

Th1 cells have long been thought to be the primary effector cells in a variety of autoimmune disorders (33). The ability to produce interferon γ (IFN-γ) and contribute to cellular defense against intracellular infections distinguishes Th1 cells from other types of cells. Innate immune cells’ production of IL-12 and natural killer cells’ production of IFN-γ allow naive CD4+T cells to overexpress transcription factors T-bet and signal transducer and activator of transcription 4 (STAT4), which in turn promotes the differentiation of naive CD4+T cells into Th1 cells (34, 35). IFN-γ is primarily secreted by Th1 cells after activation, along with tumor necrosis factor-α (TNF-α) and cytokines like interleukin-2 (IL-2). IFN-γ secreted by Th1 cells further enhances Th1 differentiation through a positive feedback loop (29) (**Figure 2**).

The most common cytokine released by Th1 cells is IFN-γ. Studies have indicated that Th1 cells cannot fully polarize upon stimulation in the absence of IFN-γ (36). IFN-γ can stabilize the Th1 phenotype and activate the T box transcription factor (T-bet), a downstream transcription target (37). On the one hand, IFN-γ can stimulate the secretion of TNF-α and enhance the immune inflammatory response, and on the other hand, it can also inhibit the secretion of IL-4 and the differentiation of Th2 cells. A study innovatively used ozone therapy to treat GLM, all patients exhibited significant symptomatic improvement simultaneously, the CD4+ T cell subsets secreting IFN-γ and TNF-α increased significantly after ozone intervention compared with those before the treatment (38), which suggests that the weakening of the immune response of Th1 cells may be one of the pathogenetic mechanisms of GLM, and that the enhancement of immune response of Th1 cells and the promotion of the secretion of IFN-γ and TNF-α in patients with GLM are able to improve the symptoms of the breast and treat GLM effectively.

In contrast, in PCM patients, Liu L et al. found that the expression of IFN-γ and interleukin-12A (IL-12A) was significantly increased in PCM breast tissues (39), and IFN-γ and IL-12A were able to activate the Th1 cell subpopulation at the same time, indicating that there may be over-activation of Th1 cells in PCM patients. Meanwhile, Liu C et al. identified an herbal drug combination using knowledge graph technology that significantly inhibited the levels of inflammatory cytokines, such as IFN-γ, TNF-α, and IL-2 secreted by Th1 cells in serum, improved clinical symptoms of PCM, and reduced the recurrence rate of PCM patients (40). This further suggests that the over-activation of Th1 cell subsets plays an important role in the pathogenesis of PCM.

#### 3.1.2 Th2

The main conditions linked to Th2 cells include allergic responses and atopic asthma. Th2 cell differentiation is dependent on the co-activation of downstream transcription factor GATA3 and signal transducer and activator of transcription 6 (STAT6), as well as cytokines such as interleukin-4 (IL-4) and interleukin-33 (IL-33) (41, 42). The primary inducer of the Th2 cell phenotype is GATA3 (43). These factors work together to stimulate the differentiation of naive CD4+ T cells into Th2 cells, which then produce interleukin-4 (IL-4), interleukin-5 (IL-5), and interleukin-13 (IL-13), triggering the type 2 inflammatory response (against type 1 immunity). In the meanwhile, Th2 cell differentiation is also favorably promoted by IL-4 (**Figure 2**).

Th2 cells produce IL-4, a cytokine that is exclusive to them. In the presence of IL-4 in the local microenvironment, naive CD4+T cells can differentiate into Th2 cells (44). Furthermore, Th2 cell activation can release IL-4, which can impede Th1 cell growth and encourage Th2 cell polarization in the Th1/Th2 immunological balance (45). Yet, not much is known about the relationship between IL-4 levels in NPM. In a single trial, the peripheral blood of GLM patients had a little lower level of IL-4 than the healthy control group, but there was no statistically significant difference between the two (46). A different study that treated PCM patients with a traditional Chinese medicine formula found that the patients’ blood levels of IL-4 decreased (40), but the levels of IL-4 did not differ between PCM patients and healthy females. Therefore, more researches are needed to further examine the expression level of IL-4 in GLM and PCM.

The IL-33 receptor (IL-33R) is a heterodimer made up of the IL-1 receptor accessory protein (IL-1RAcP) and the tumorigenicity 2 receptor (ST2) (47, 48). IL-33 is a member of the IL-1 family. By attaching to its receptors ST2 or IL-1RAcP, IL-33 can increase the secretion and chemotaxis of Th2 cytokines, hence enhancing type 2 inflammatory and immunological responses (49, 50). It has been established that the IL-33/ST2 axis is crucial in several inflammatory and autoimmune disorders (49). The bait receptor of IL-33 is sST2, which is the soluble version of ST2. when sST2 binds to IL-33, it can inhibit IL-33 signaling and inhibit the proliferation and differentiation of Th2 cells (50). It was found that the level of IL-33 in the GLM group was significantly higher than that in the group of breast cancer (BC) patients and the normal control group, but the level of sST2 in the GLM group was significantly lower than that in the BC group (13). The higher level of IL-33 in GLM patients contributes to the proliferation and differentiation of Th2 cell subpopulations, leading to enhanced immune-inflammatory responses. Additionally, the lower level of sST2 suggests that the suppression of IL-33 in GLM patients is less pronounced compared to BC patients. This indicates a higher overall activity of the IL-33/ST2 signaling pathway in GLM patients, potentially resulting in a significant proliferation of Th2 cells.

However, another study found that although both GLM patients and breast cancer patients had higher serum IL-33 than healthy women, there was no significant difference in serum IL-33 levels between the GLM group and BC group (51). Two other studies used flow cytometry and enzyme-linked immunosorbent assays (ELISAs) to detect IL-33 levels in blood samples from GLM patients and normal subjects, respectively, and showed no significant differences (52, 53). Despite variations in test groups and measuring techniques, conflicting findings suggest that the question of whether GLM patients exhibit hyperactivated type 2 immune inflammatory responses remains open. Further exploration with additional assays and larger sample sizes is needed to clarify this issue.

#### 3.1.3 Th17

Th17 cells have been implicated in several human autoimmune disorders in recent years (54). When interleukin-6 (IL-6) is present in an inflammatory environment, transforming growth factor-β (TGF-β) can synergistically promote the differentiation of Th17 cells. Interleukin-23 (IL-23) then promotes further differentiation into Th17 cells and keeps Th17 cells maturing steadily (55). Concurrently, RORγt and RORα transcription factors play a core role in the differentiation process of Th17 cells. Signal transducer and activator of transcription 3 (STAT3) perceives and transduces signals from cytokines, and induces polarization of Th17 cells under the induction of transcription factors RORγt and RORα (56). Th17 cells can secrete a variety of cytokines upon differentiation and maturation, including interleukin-17A (IL-17A), interleukin-17F (IL-17F), interleukin-21 (IL-21), and interleukin-22 (IL-22) (56) (**Figure 2**).

IL-17 is a specific cytokine secreted by a subpopulation of Th17 cells, with IL-17A and IL-17F being the most extensively studied isoforms. IL-17 acts as a pro-inflammatory factor, inducing neutrophil recruitment, enhancing inflammatory response, and leading to tissue damage (57, 58). Its over-activation has been implicated in autoimmune diseases such as rheumatoid arthritis and psoriasis (59–61). Cakir et al. reported no significant difference in IL-17A levels in GLM patients when compared to healthy controls (53). However, two other studies found that IL-17 levels in GLM patients were significantly higher than those in healthy controls (46, 52), suggesting the presence of an immune-inflammatory response with over-activation of Th17 in patients with GLM. In contrast, the researchers found that there was no statistically significant difference in the expression levels of IL-17A in the breast tissue of PCM patients compared to healthy individuals (39). Thus, patients with GLM may present with an immune-inflammatory response characterized by Th17 cell over-activation, while patients with PCM do not.

IL-6 is a vital driver of Th17 cell differentiation that plays a significant role in immune inflammation. IL-6 can stimulate the production of acute-phase proteins, including C-reactive protein (CRP) (62). Additionally, IL-6 collaborates with TGF-β to promote the differentiation of Th17 cells, while simultaneously inhibiting the differentiation of Treg cells (63). IL-6 is a crucial pro-inflammatory factor. Elevated levels of IL-6, neutrophil, and CRP were observed in the blood of patients with GLM compared to those with benign breast tumors. Moreover, a positive correlation was found between IL-6 levels and the values of neutrophil, CRP, IL-17, and TNF-α (64). Another study revealed a correlation between the severity of GLM inflammation and elevated IL-6 levels, which were notably higher in patients with severe GLM compared to those with mild and moderate disease. Furthermore, increased serum IL-6 levels were often associated with a prolonged period of remission in GLM (65). Therefore, we speculate that in GLM patients, an immunological response driven by IL-6 may overactivate Th17 cells, resulting in tissue damage and acute inflammation.

Furthermore, there is a tight relationship between Th17 cells and IL-23. IL-23 plays a crucial role in sustaining Th17 cell activity and encouraging Th17 cell development (55, 66). Research has demonstrated that GLM patients exhibit significantly higher serum levels of IL-22 and IL-23 compared to control individuals without a history of breast disease (67). However, the study has certain limitations and cannot prove that the increase in IL-23 and IL-22 is mediated by Th17 cell activation rather than other immune cells. In addition, it is important to acknowledge the relatively small sample size in this study, thus warranting further validation in larger cohorts and populations. Nevertheless, the concurrent increase in IL-22 and IL-23 implies the potential significance of the Th17 cell subset in the pathogenesis of GLM.

### 3.2 Regulatory T cells

Regulatory T cells (Treg cells) primarily differentiate from CD4+ T lymphocytes in the thymus and play a crucial role in maintaining immune homeostasis and self-tolerance through immune response suppression and regulation (68). FOXP3, a crucial transcription factor that controls Treg cell formation and function, enables Treg cells the capacity to suppress activity. It’s also widely recognized as a major marker for identifying Treg cells (69, 70). Furthermore, the differentiation of Treg cells is based on the stimulation of IL-2 and TGF-β. IL-2/STAT5 pathway and high levels of TGF-β concurrently promote the expression of the FOXP3 gene and contribute to the differentiation of Treg cells (71, 72). Activated Treg cells secrete substantial quantities of inhibitory cytokines, including TGF-β and IL-10 (73), which effectively suppress the immune response (**Figure 2**).

A study employed flow cytometry to investigate the expression of FOXP3 in the Treg cell subpopulation in patients with GLM. The Treg cells were labeled with FOXP3, and the analysis revealed significantly reduced expression of FOXP3 in the GLM group compared to the normal group during both the active and remission phases (74). Similarly, another study reported lower levels of Treg cells in patients during the active phase (31). Based on the extant research pertaining to Th17 cells and GLM, it is plausible to postulate the existence of a Th17/Treg immune imbalance in patients afflicted with GLM. Specifically, this imbalance manifests as an over activation of Th17 cell-mediated immune responses, and an inadequate immunosuppression exerted by Treg cells.

Contrary to these findings, alterations in cytokine levels presented a contrasting conclusion. TGF-β, secreted by Treg cells, promotes their differentiation. Additionally, IL-2 binds to the IL-2 receptor (IL-2R), expressed during Treg cell differentiation, thereby fostering Treg cell differentiation and immunosuppressive function (75). Li Y et al. discovered elevated levels of IL-2 in 23.7% of 333 GLM patients (76). Another study demonstrated elevated levels of both TGF-β and IL-2 in GLM patients compared to the control group, and it observed a notable decrease in TGF-β-secreting Treg cells following effective therapeutic interventions (38). We recognize the limitations of the studies mentioned above. Various immune cells can secrete the same inflammatory factors, and further in-depth study is required to understand how distinct immune cells interact with one another in the immunological microenvironment.

Although IL-10 is not specific to Treg cells, it acts as an anti-inflammatory cytokine that not only suppresses the release of various pro-inflammatory cytokines but also inhibits the activation and function of immune cells such as Th1 and Th17, thus performing a similar function as Treg cells in suppressing excessive immune responses. In a prospective study, lower concentrations of IL-10 were detected in the blood of patients with GLM than in controls using flow cytometry (53). In contrast, another study employing ELISA found higher levels of IL-10 in patients with GLM than in controls (46). The possible reasons for this discrepancy could be differences in testing methods and populations, and the study did not explicitly specify whether the GLM patients were in the active or remission stages of the disease. Additionally, the varied course of the disease in each patient could lead to differences in study outcomes.

### 3.3 Cytotoxic T lymphocytes

Cytotoxic T lymphocytes (CTLs), also known as CD8+ T cells due to their expression of CD8 receptors on the cell surface, are activated when major histocompatibility complex-1 (MHC-I) molecules present endogenous antigens to the TCRs on their surface (77). Upon activation, CTLs release perforin and granzymes, leading to lysis and apoptosis of target cells. Additionally, CTLs can secrete cytokines such as TNF-α and IFN-γ (78), inducing apoptosis and inflammation (**Figure 2**).

In a study involving immunohistochemical staining of lesion tissues from GLM patients, the diffuse presence of CD8 lymphocytes in GLM lesion tissues suggested the likely involvement of CTLs in the development of GLM (14). The study revealed a significant increase in CTLs among patients in the active phase of GLM compared to both healthy volunteers and patients in the remission phase of GLM (31). Therefore, the increased presence of CTLs leads to a state of immune activation in the body, which may be a critical factor contributing to the active phase of GLM. Another study reached a similar conclusion (30). Cabioglu et al. employed ozone therapy for GLM treatment and observed a significant increase in TNF-α-secreting CTLs and a notable decrease in IL-10-secreting CTLs in the peripheral blood of GLM patients after ozone (38). These findings suggest that different cytokine secretion patterns occur in CTLs during GLM treatment; however, further extensive research is required to validate and elucidate the specific patterns of these changes.

Additionally, CTLs can trigger programmed apoptosis in target cells by interacting through Fas ligand (FasL) on their surface with Fas proteins on the surface of the target cells (79). This interaction activates the caspase-8 cascade reaction, which in turn causes apoptosis to specifically occur in target cells that contain antigens (80). Zuo XM et al. discovered an upregulation of activated Gasdermin D (GSDMD) and Cysteinyl aspartate-specific proteinase-1 (caspase-1) expression in the lesional tissues of GLM patients, accompanied by evident cellular pyroptosis observed using transmission electron microscopy (81). These findings suggest a close association between GLM and caspase-1-mediated pyroptosis and inflammatory response. Nonetheless, caspase-8 in mammals is a member of the apoptotic caspase family and is primarily engaged in apoptosis and autophagy (82). A study has shown that *Staphylococcus aureus*, a cause of acute mastitis, can induce apoptosis in primary bovine mammary epithelial cells and activate caspase-8 signaling through Fas and Fas related death domains (83). However, the relationship between NPM and Fas/FasL mediated caspase-8 activation, cell apoptosis, and autophagy is not yet covered in any publications, and this is a worthwhile area for future investigation.

### 3.4 B cells

B cells arise from hematopoietic stem cells in the bone marrow and express the surface molecule CD20 (84). The histological analysis of PCM revealed a significant infiltration of plasma cells (9, 85), which are derived from B cells. This suggests that B cell-mediated immune-inflammatory responses play a dominant role in PCM. Therefore, a study classified non-lactating mastitis patients into GLM and PCM according to the results of the antibody staining of CD3 and CD20 in their breast lesion tissues (6). However, B cells, expressing a signaling receptor composed of the BCR complex consisting of CD79a and CD79b (86), also participate in the development of GLM. This was supported by the detection of CD79a lymphocytes in GLM tissues (14, 87), also indicating the involvement of B cells in the pathogenesis of GLM.

Upon binding to specific antigens, some activated B cells differentiate into plasma cells. The infiltration of a large number of plasma cells is a characteristic feature of PCM (85). The differentiation and function of plasma cells are influenced by various cytokines, with IL-6 being particularly important (88, 89). The IL-6 receptor comprises two subunits: the binding subunit (IL-6 receptor) and the signaling subunit (gp130). When IL-6 binds to the IL-6R on the cell surface, it phosphorylates the Janus kinase (JAK), which in turn phosphorylates the tyrosine residues of gp130, providing a binding site for STAT3 (90, 91). The phosphorylated STAT3 enters the nucleus for transcriptional activation, thereby influencing the functional phenotype of the cell. B-cell lymphoma 2 (Bcl-2), a downstream target gene of STAT3, plays a role in the regulation of apoptosis (92) (**Figure 1**). Overexpression of Bcl-2 has been shown to inhibit apoptosis of plasma cells (93), and Bcl-2 inhibitors have demonstrated efficacy in the treatment of certain tumor diseases characterized by over proliferation of plasma cells, such as multiple myeloma (94) (**Figure 1**).

Previous studies have demonstrated a strong correlation between PCM and the activation of the IL-6/JAK/STAT3 signaling pathway, as well as the upregulation of Bcl-2 expression (95) (**Figure 1**). Specifically, breast tissues from PCM patients exhibited significantly higher levels of IL-6 and Bcl-2 compared to patients with acute mastitis or the control group. Moreover, there was a notable positive correlation between IL-6 and p-STAT3 staining (95). Notably, PCM patients with inverted nipples displayed significantly elevated IL-6 expression in comparison to other PCM patients (95) (**Figure 1**). A study used IL-6 injection into the mammary glands of mice that had received normal mammary tissue homogenate. Consequently, the mouse mammary gland exhibited characteristics resembling human PCM. Notably, the mammary glands of the PCM mice revealed a substantial increase in IL-6/JAK2/STAT3 signaling activity in CD138+ plasma cells and a high expression of Bcl-2 (96). This observation indicates the successful activation of the IL-6/JAK2/STAT3 pathway and the establishment of the PCM mouse model. In response to the elevated IL-6 levels observed in PCM, treatment with Sinomenine hydrochloride at a dose of 100 mg/kg significantly attenuated plasma cell infiltration in the mammary glands of PCM mice (97). Additionally, it downregulated the expression of factors associated with the IL-6/JAK2/STAT3 pathway and Bcl-2, thus demonstrating its potential for treating PCM in mice. These findings provide valuable insights for future research on PCM, particularly regarding the exploration of IL-6/JAK2/STAT3 pathway inhibitors and Bcl-2 inhibitors as potential therapeutic options.

Furthermore, plasma cells can produce immunoglobulins (IgG, IgM, etc.) and regulate the immune response (98). Histopathological analysis revealed that the expression of IgG and IgM in local GLM breast tissue was within the normal range, as only a small number of plasma cells were present (14, 52). IgG4 is an immunoglobulin isoform secreted by plasma cells. However, IgG4 expression levels were found to be significantly higher in patients with GLM nipple inversion than in patients without nipple inversion (15), suggesting that ductal occlusion, damage to the ductal endothelium, and release of localized autoantigens can lead to localized immune responses and elevated IgG4 in GLM mammary glands. Currently, IgG4-related sclerosing mastitis is recognized as a distinct disease from GLM and PCM, characterized by massive infiltration of IgG4+ plasma cells in breast tissue (99, 100). There is a massive infiltration of plasma cells in PCM as well. Nevertheless, there are certain imperfections in the research in this area, including the lack of studies on the expression levels of distinct immunoglobulins in PCM breast tissues and the variations in immunoglobulin expression in different NPM types. These research areas deserve further in-depth investigation from researchers in the future.

## 4 Roles of intrinsic immune cells and molecules in NPM

### 4.1 Macrophages

Macrophages’ role in mediating the local immune response is crucial to the pathophysiology of GLM. Macrophages support tissue fibrosis and the adaptive immune response in addition to helping to create the dense packing structure of granulomas (101). The characteristic pathological manifestation of GLM is the formation of inflammatory granulomas centered around the mammary lobules (1). The most recent single-cell sequencing data highlights the role of macrophages in granulomatous diseases by revealing that Th17.1 cells that produce purines, fibroblasts with inflammatory and tissue remodeling phenotypes, and macrophages undergoing metabolic reprogramming are important players in the formation of skin granulomas (102).

The formation of inflammatory granulomas consists of a series of procedures, the central process of which is the activation and aggregation of macrophages, along with the recruitment of other immune cells, and the production of a variety of structural changes including epithelioid transformation, fusion into multinucleated giant cells, and phagocytosis of oxidized lipids to form foam cells (103). The most common cytological feature of GLM is the presence of epithelioid histiocytes (11, 104), which are very likely to be transformed from macrophages. Moreover, multinucleated giant cells and foam cells are common cells detected histopathologically in GLM (16). Most importantly, macrophage infiltration and activation of M2-type macrophages were reported in both PCM and GLM breast tissues in the current study (15).

Under a variety of conditions, including pathogen invasion, tissue injury, and metabolic problems, mature macrophages undergo phenotypic and morphological differentiation, a process known as macrophage polarization. The two primary types of macrophages are classic/inflammatory activated macrophages (M1 macrophages) and replacement/wound healing activated macrophages (M2 macrophages) (105). M1 macrophages have a pro-inflammatory phenotype and strong phagocytic and cytotoxic abilities, capable of secreting pro-inflammatory cytokines such as IL-6, IL-12, IL-1β and TNF-α and chemokines such as CCL2 and CCL5, and recruit other immune cells to reach the infected site to maintain their activation. It was found that the values of TNF-α in the peripheral blood of patients with GLM were lower than those of healthy controls (53), while Zhou Y et al. found that serum levels of TNF-α were higher in patients with GLM than in patients with benign breast tumors (64). The diametrically opposed results of the two studies may be attributed to differences in the control populations, namely healthy individuals versus patients with benign breast tumors, on the one hand, and on the other hand, to the possibility that the level of TNF-α may be influenced by other immune cells, such as Th1 cells. Interestingly, a different study concentrated on the chemokines released by macrophages and discovered that the Nuclear Factor-κB (NF-κB) inflammatory pathway can be activated by M1 macrophages through overexpression of the chemokine CCL-5, which may be a key mechanism in the pathophysiology of GLM (106). M2 macrophages mainly play a role in inflammation resolution and tissue repair pathways and can produce cytokines such as IL-10 and TGF-β, which can participate in Treg cell-dominated immune responses and suppress inflammatory responses (107). However, the presence of M2 macrophages in GLM and the changes in their associated factors are not known, which requires further studies by scholars in the future.

Furthermore, Nod2 (CARD15) is a protein exclusive to macrophages, and recent studies have linked NOD2 gene alterations to Crohn’s disease (108). Defects in the NOD2 gene can trigger the Th1 response, which is mediated by Toll-like receptor 2 (TLR2) and the NF-κB signaling pathway (109). According to a study, a GLM patient with a *Corynebacterium Klebsiella* infection experienced neutrophil dysfunction due to a mutation in the NOD2 gene (110). However, the present study fell short in demonstrating a correlation between the NOD2 gene and macrophages, or elucidating whether its mutations contribute to the progression of GLM via specific immune-mediated pathways, thereby necessitating further investigative endeavors.

### 4.2 Neutrophils

One of the body’s most significant phagocytic cells is the neutrophil (111). Its role in innate and adaptive immunity is primarily mediated by three pathways: degranulation, phagocytosis, and neutrophil extracellular trap (NET) (112). Furthermore, it has the ability to release pro-inflammatory cytokines and chemokines (111). Neutrophils react to endogenous ligands in aseptic inflammation, especially damage-associated molecular patterns (DAMPs) (113). Dead cells are released into the surrounding tissues through DAMPs, which sets off a series of pro-inflammatory signals that encourage the activation and recruitment of neutrophils and other immune cells to the site of tissue damage, ultimately resulting in inflammation (114).

Neutrophil counts are commonly used in clinical practice to assess the inflammatory state and immune function of the body. Neutrophil counts, neutrophil percentages, and neutrophil-to-lymphocyte ratios in the peripheral blood of patients with GLM have been reported to be significantly elevated compared with the normal range (31, 52, 64). Another study has shown that the preoperative neutrophil to lymphocyte ratio (NLR) in GLM patients is significantly correlated with the probability of postoperative recurrence. The higher the NLR, the greater the probability of GLM recurrence (115).

Neutrophils release NET, a DNA structure that is adorned with nuclear, granular, and cytoplasmic proteins that have the ability to both inhibit and eliminate pathogens (116). During chronic inflammation, uncontrolled released NETs can lead to cytotoxicity and worsen tissue damage (117). Currently, a number of autoimmune disorders have reported their link with NETs (118). Although neutrophils have been shown to be an effective measure of NPM severity, it is still unknown if NET plays a role in the pathophysiology of PCM or GLM. This seems like a great promising avenue for exploration.

### 4.3 Natural killer cells

Through the release of cytotoxics or recognition of chemicals on the surface of target cells, natural killer cells (NK cells) offer quick non-specific killing against a variety of diseases (119). It can also participate in other related pathways and create cytokines like IFN-γ to control adaptive immune responses (120). NK cells can activate dendritic cells and trigger more potent immunological responses of CTLs due to their interaction with other immune cells. Additionally, they have the ability to produce IFN-γ, which facilitates antigen presentation, processing, and antigen-specific T cell polarization in the direction of Th1 (121).

Emsen et al. reported that both the count and percentage of NK cells were elevated in GLM patients compared to healthy controls (30). Kong et al. identified that NK cells may be important cells in distinguishing between GLM and PCM, as they found that the number of NK cells in GLM patients increased with disease progression from 2 weeks to 3 months, then reaching a peak thereafter, whereas this change was not observed in patients with PCM (15). However, not much is known about the interaction between natural killer cells and other immune cells; hence, future researchers ought to focus more on these topics.

### 4.4 Complement

**Figure 3:**
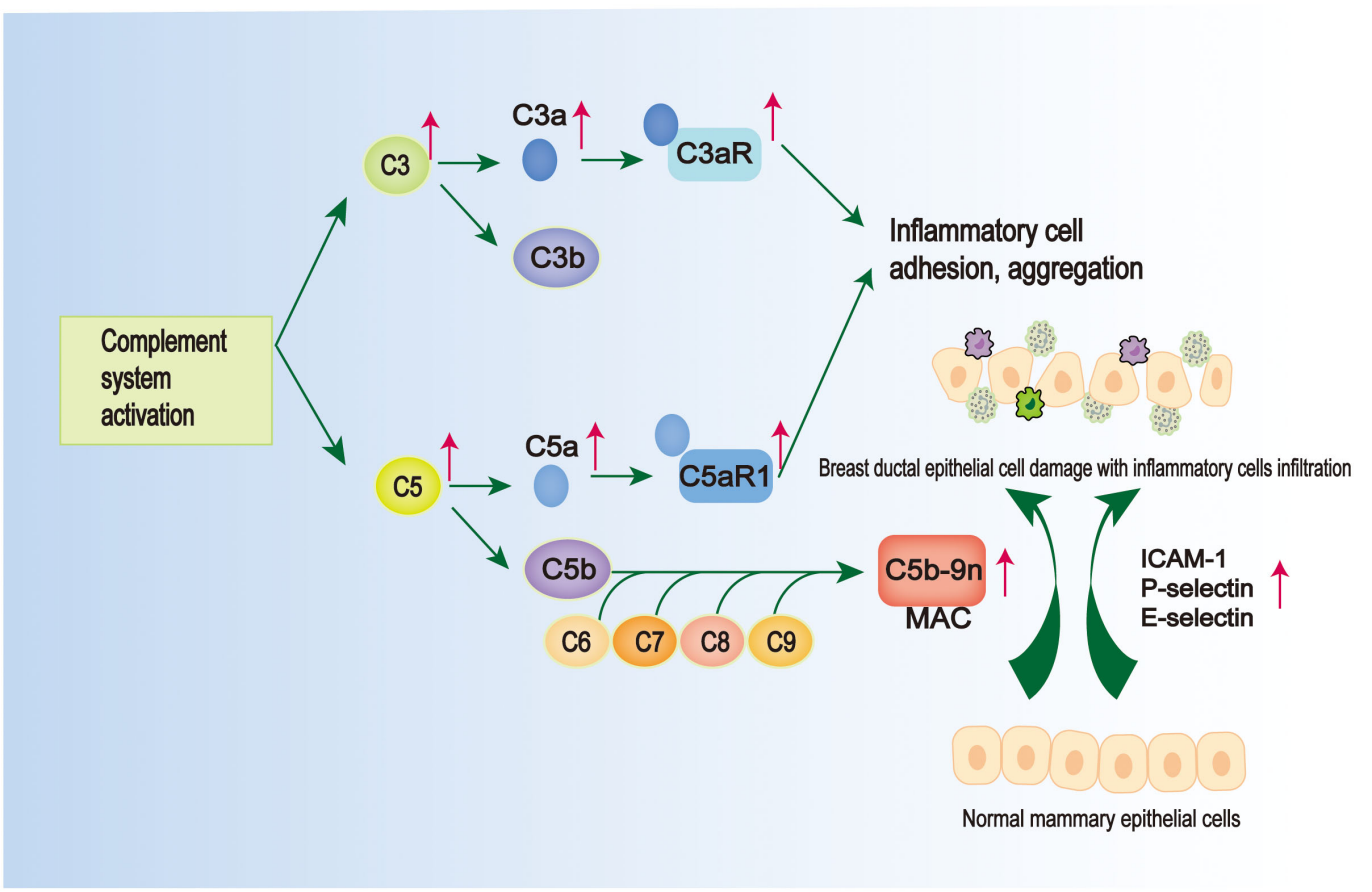
Pathological mechanisms common to GLM and PCM. There is activation of the complement system in GLM and PCM. On the one hand, complement activation can lead to the formation of membrane attack complex (MAC), which is located on the epithelial membrane cells and leads to the damage of mammary ductal epithelial cells (127); on the other hand, there are elevated levels of C3/C3a-C3aR and C5/C5a-C5aR1 in their tissue samples, and the intercellular adhesion factors (ICAM-1, E-selectin, and P-selectin) are elevated, which can promote inflammatory cell adhesion and aggregation (128).

The complement system is an important part of the intrinsic immune system (122). Its activation facilitates the cleavage of complement C3 into C3a and C3b (123). This process not only enables the formation of pathogen-complement complexes, enhancing phagocytosis by macrophages (124), but also leads to the assembly of other complement proteins, resulting in the formation of the C5 convertase enzyme. The C5 convertase enzyme, in turn, converts C5 into C5a and C5b (125). Subsequently, C3a and C5a bind to their receptors C3aR and C5aR1, respectively, triggering a series of biological responses such as vasodilatation, inflammatory response, tissue damage, and also chemotaxis, which promotes the aggregation of various inflammatory cells, such as macrophages, neutrophils, etc. (126). While C5b binds to the surface of target cells, it sequentially binds to C6, C7, C8 and C9 molecules to form membrane attack complex (MAC), which mediates the target cell lysis effect. It was found that C5a was detected to be significantly elevated in GLM lesion tissues compared to the normal range, and the value of complement C3 also belonged to the high side of the normal range (52), suggesting that there may be a mechanism of complement activation in the pathogenesis of GLM (**Figure 3**). This has since been confirmed by Li XQ et al. with a more detailed study, who found elevated levels of C3/C3a-C3aR and C5/C5a-C5aR1 in PCM and GM tissue samples (128) (**Figure 3**).The binding of C3a and C5a to their corresponding receptors promotes leukocyte-endothelial cell interactions and adhesion to inflammatory cells (**Figure 3**).

Notably, intercellular adhesion molecule 1 (ICAM-1) is highly induced in various immune cells and can regulate leukocyte rolling and adhesion interactions with vascular walls, promoting the clearance of apoptotic cells (129). Selectin adhesion molecules (E-selectin, P-selectin) can mediate the rolling of white blood cells on activated endothelium, which is a prerequisite for the accumulation of white blood cells at the site of inflammation (130). It has been observed that the expression and immunoreactivity of ICAM-1 are significantly upregulated in ductal endothelial cells and epithelial cells in PCM patients (131). This elevated expression prompts the accumulation of inflammatory cells, including macrophages, at the ductal epithelium and subsequently leads to the generation of excessive immune responses (131) (**Figure 3**). In a recent study conducted by Zhang HJ et al., it was observed that P-selectin, E-selectin, and ICAM-1 exhibited high expression levels in various immune cells, including macrophages, in both PCM and GLM (127). Additionally, the study identified the presence of mammary ductal epithelial damage in both PCM and GLM, accompanied by a significant increase in MAC located in the cell membrane of mammary ductal epithelial cells (127) (**Figure 3**). Although the study did not investigate the mechanism of complement in MAC formation or the role of complement in the pathogenesis of GLM, these findings suggest that the aggregation of diverse immune-inflammatory cells, including macrophages and neutrophils, which leads to MAC-mediated injury of breast ductal epithelial cells, may serve as a critical mechanism in the development of GLM and PCM.

## 5 Crosstalk between NPM and other immune factors

### 5.1 Prolactin

**Figure 4:**
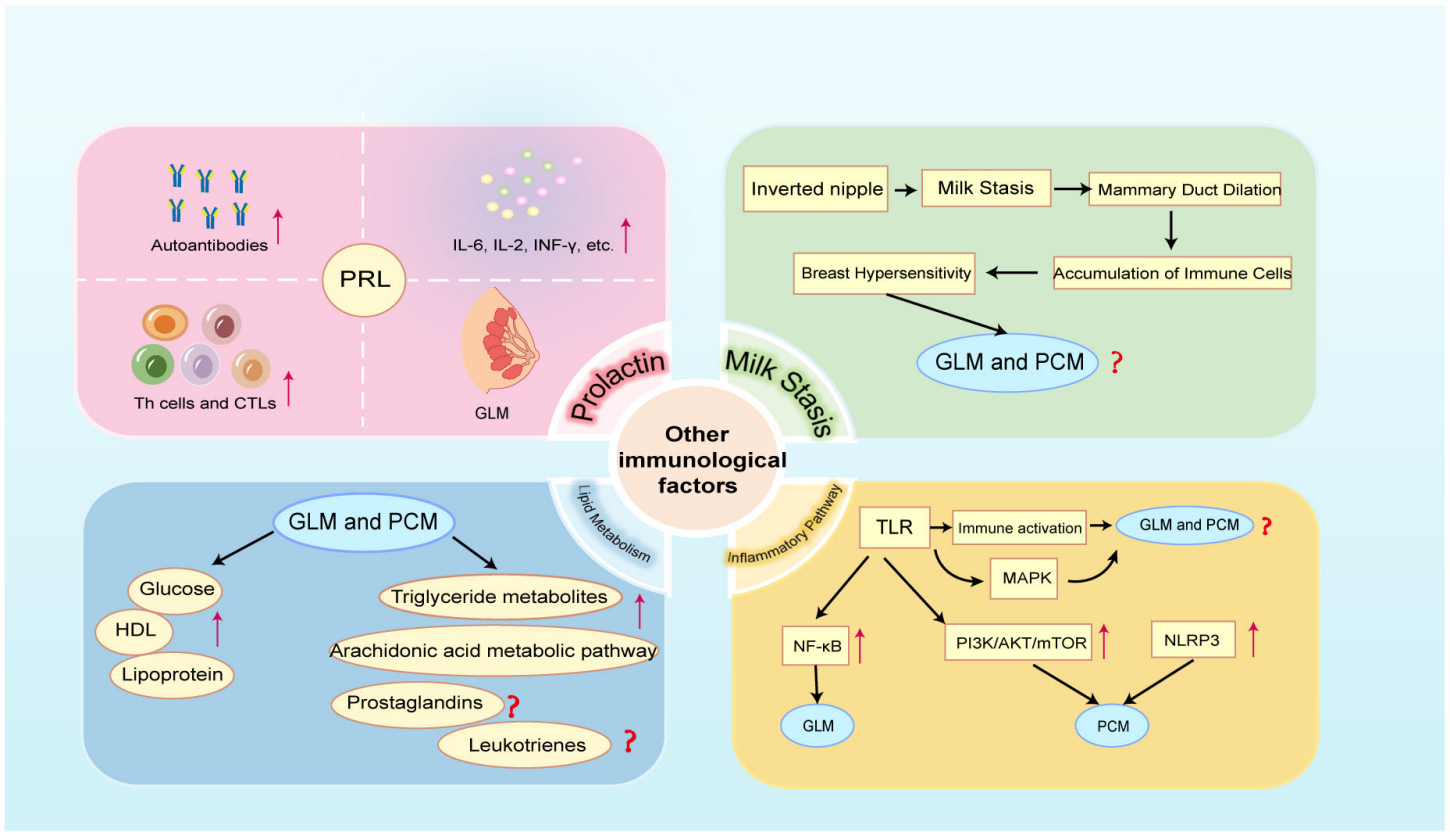
Crosstalk between other immune factors and NPM (GLM and PCM). PRL, prolactin; HDL, high-density lipoprotein; TLR, Toll-like receptor; MAPK, mitogen-activated protein kinase; NF-κB, nuclear factor-κB; PI3K/AKT/mTOR, phosphatidylinositol 3- kinase/protein kinase B/mammalian target of rapamycin; NLRP3, NOD-like receptor protein 3.

The observation that women of childbearing age had a higher incidence of GLM lends credence to the theory that specific hormones, such as prolactin (PRL) in women, trigger the development of GLM (**Figure 4**). It has been established that hyperprolactinemia is the cause of GLM (132, 133). In addition to being a hormone, PRL is a cytokine that regulates immunological responses. Hyperprolactinemia can powerfully activate both innate and adaptive immune responses, and it is linked to the pathophysiology of several autoimmune disorders (134). Reports state that PRL is directly correlated with T and B cell counts and can regulate Th cells and CTLs maturation via IL-2 receptors (135, 136). In addition, it can control both type 1 and type 2 immune responses as well as associated cytokines like IL-2, IL-6, and INF-γ (134, 137, 138). In patients with hyperprolactinemia, several autoimmune antibodies have even been found (139). Unfortunately, present research has not explored the precise mechanism by which hyperprolactin contributes to the start of GLM, instead focusing only on the fact that hyperprolactin is the cause of GLM. Further researches in this field ought to be undertaken by academics in the future.

### 5.2 Local hypersensitivity caused by milk retention

A large proportion of PCM and GLM patients have inverted nipple deformity (140, 141) (**Figure 4**). When accompanied by hyperprolactinemia during non-lactation, inverted nipples are bound to cause milk to accumulate in the mammary ducts. Studies have demonstrated that the alveolar distension caused by milk retention raises cytokine production and activates STAT3, which sets off a cascade of lysosomal-mediated cell death (142, 143). Moreover, breast epithelial cell death and the rise in intracellular Ca2+ are caused by milk retention (144). Insufficient emptying of the breast can cause dilatation of the breast alveoli, which raises pressure in the mammary ducts and causes the epithelial cells inside the ducts to become loosely connected (145). When the milk reaches the lobular stroma, it comes into contact with a multitude of immune cells, primarily macrophages, as well as a profusion of immunoglobulins and cytokines that can cause localized primary aseptic inflammation (146).

### 5.3 Disordered lipid metabolism

Numerous lipids, such as glycerophospholipids and lipoproteins, exhibit pro- and anti-inflammatory and immunomodulatory characteristics (147). Of them, high-density lipoprotein (HDL) possesses a variety of immunomodulatory and anti-inflammatory characteristics because of its capacity to transport bioactive lipids and antioxidants (148). By controlling the outflow of cellular cholesterol, it controls the activation of immune cells (149). As a result, lipid metabolism and immunological inflammation are closely related.

A retrospective study comprising 284 NPM patients revealed that the lipoprotein and blood glucose levels in the NPM group were significantly higher than those in the normal group, while HDL levels were significantly lower than those in the normal group (5) (**Figure 4**). This is the first study to suggest that lipid metabolism-related indicators could be crucial to the development and treatment of NPM. A recent study of untargeted lipidomics in breast tissue samples from patients with NPM showed that lipid metabolism is indeed disturbed in patients with NPM, with triglyceride metabolites being the predominant differential metabolite and arachidonic acid metabolism being the main pathway contributing to the differences in lipid profiles (150)(**Figure 4**). Abnormal triglyceride metabolism triggers oxidative stress, excessive lipid accumulation, immune cell activation and inflammation. Similarly, arachidonic acid can synthesize lipid metabolites such as prostaglandins and leukotrienes, which trigger strong inflammatory responses, through the cyclooxygenase pathway, the lipoxygenase pathway, and the cytochrome P450 pathway (151). Based on the above research foundation, future studies should further focus on the specific molecular mechanisms of lipid metabolism occurring in NPM and its crosstalk with the immune system.

### 5.4 Related signal pathways

Toll-like receptors (TLRs), as a typical pattern recognition receptor (PRR), can recognize the molecular patterns of pathogens and activate immune cells to clear them (152). TLR signaling can directly or indirectly regulate a wide range of immune cells dominated by T cells (153). TLR2/4 on dendritic cells (DCs) activate CD4+ T cells by recognizing their respective ligands, facilitating the antigen presentation process, and upregulating co-stimulatory molecules on the surface of DCs (153). In addition, TLR3 and TLR9 also show a strong ability to induce CD8+ T cell activation *in vivo (* 154). Notably, CD4+ T cells express TLR2 upon antigenic stimulation, and TLR2 signaling directly promotes the proliferation of Th1 cells, which in turn promotes the secretion of inflammatory factors (155). Interestingly, it was shown that TLR8-dependent signaling also attenuates the immunosuppressive function of Treg cells (156). Given the regulatory roles between TLRs and a wide range of immune cells, their importance in the immune system cannot be overstated. Future studies should aim to deeply explore the differences in TLR expression, immune cells, and cytokines between NPM patients and healthy individuals. Through comparative analysis, we can hope to find more precise treatments for NPM and bring breakthroughs in the immunology of NPM.

TLRs can directly regulate immune cells and also induce excessive immune responses by activating relevant inflammatory pathways. There are 10 human TLRs (TLR1–10), and different TLRs recognize different extracellular ligands. All TLRs activate mitogen-activated protein kinase (MAPK) and NF-κB signaling pathways and enhance the secretion of chemokines and inflammatory factors, thereby inducing excessive immune inflammatory responses (157) (**Figure 4**). Activation of the NF-κB inflammatory pathway has been shown to exist in GLM (**Figure 4**), but unfortunately, this study did not delve into the specific relationship between TLR and NF-κB in GLM (106). This area certainly deserves further exploration in future studies. It is even more unfortunate that no current studies are addressing the existence of the activation state of TLR and MAPK signaling pathway in NPM. Future studies should delve into the potential role of TLR and MAPK signaling pathways in the pathogenesis of NPM to provide new perspectives for the development of new therapeutic strategies.

Additionally, pathogens activate multiple inflammation-related signaling pathways downstream through TLR4, including the phosphatidylinositol 3-kinase/protein kinase B/mammalian target of rapamycin (PI3K/AKT/mTOR) signaling pathway (158) (**Figure 4**). At the site of inflammation, PI3K, which is abundantly expressed in white blood cells, facilitates the recruitment and activation of innate immune cells, and it is crucial for the growth, differentiation, and operation of T and B lymphocytes (159). The mTOR signaling pathway is essential for thymic T cell growth and differentiation, as well as for controlling the migration of Tfh cells and Treg cells (160, 161). Consequently, there is an unambiguous interaction between different immune cells and the PI3K/Akt/mTOR pathway. As mentioned previously, there is an over-activated IL-6/JAK2/STAT3 signaling pathway in PCM (96). JAK kinase is an activator of the PI3K/AKT signaling pathway, and phosphorylated JAK activates PI3K, which in turn activates its downstream cascade (**Figure 1**). Previous research has demonstrated that as compared to normal tissue, PCM tissue had higher levels of p-AKT and p-mTOR. This suggests that the PI3K/Akt/mTOR signaling pathway, which is active, may be involved in PCM and that exosomes may mediate this process (23). Nevertheless, whether PI3K pathway inhibitors are therapeutic for PCM is not yet apparent in this investigation. Thus, researchers should do more to understand the precise regulatory mechanism of the PI3K/AKT/mTOR pathway in NPM.

Furthermore, damage-associated molecular patterns (DAMPs) are also involved in the inflammatory process; they drive innate immune responses, activate inflammasomes, initiate immunological responses, and encourage tissue regeneration and damage repair (162). It has been established that the NOD-like receptor protein 3 (NLRP3) inflammasome is linked to a number of inflammatory immunological disorders (163). In a recent study, NLRP3 inhibitor was used to treat PCM in mice, and it was shown that the NLRP3 inhibitor may treat PCM in mice by boosting the quantity and activity of bone marrow-derived suppressor cells (MDSCs) (164) (**Figure 4**). Fortunately, this study demonstrates the activation of NLRP3 inflammasomes in PCM mice, providing a new perspective for subsequent research. Of course, more thorough investigations are required to fully understand the particular regulatory processes in action.

## 6 Conclusion and outlook

In conclusion, our research shows that the pathophysiology of GLM and PCM is related to multiple immune variables, including immune cells, connecting cytokines, prolactin, hypersensitivity reactions driven by milk stasis, lipid metabolic disorders, and immune inflammation associated pathways. Both innate immunity and adaptive immunity play different roles in the development of GLM and PCM, and during the process, local damage to mammary ductal epithelial cells happens, which intensifies immunological inflammatory responses in breast tissue.

Specifically, alterations in immune cells and associated cytokines/molecules were observed in both GLM and PCM patients, when compared to healthy individuals, patients with benign breast tumors, or NPM patients after treatment (**Table 1**). By summarizing the available literature, we found the following: in terms of macroscopic immune cells, there may be a decrease in Th cells and Treg cells and an increase in CTLs, macrophages, neutrophils, and NK cells in GLM, whereas there is an increase in B cells and macrophages in PCM; in terms of microscopic cytokines, there may be a suppression of Th1 cells and an increase in Th17 cells in GLM, while there may be overactivation of Th1 cells in PCM; furthermore, activation of the complement system was present in both GLM and PCM, as evidenced by increased expression levels of C3/C3a-C3aR, C5/C5a-C5aR1, and membrane attack complex (MAC).

**Table 1:** Changes in immune-related cells and molecules in GLM and PCM in different studies.

Types of immune cells		Function	Changes in GLM patients	Changes in PCM patients	Source	Associated Cytokines/Molecules	Changes in GLM patients	Source	Changes in PCM patients	Source
T helper cells (Th cells)	Th1 cells	Activating macrophages and cytotoxic T-cells, mediating cellular immunity	↓	–	(30, 31)	IFN-γ	↓	(38)	↑	(39, 40)
						TNF-α	↓		↑	(40)
						IL-2	–	–	↑	
						IL-12A	–	–	↑	(39)
	Th2 cells	Promoting B cell differentiation and mediating humoral immunity				IL-4	No statistical difference	(46)	↑	(40)
						IL-33	↑	(13, 51)	–	–
							No statistical difference	(52, 53)	–	–
	Th17 cells	Fighting bacterial and fungal infections				IL-17	↑	(46, 52)	No statistical difference	(39)
						IL-6	↑	(64, 65)	–	–
						IL-23	↑	(67)	–	–
Cytotoxic T lymphocytes (CTLs)		Killing target cells that phagocytose pathogens	↑	–	(30, 31)	TNF-α	↓	(38)	–	–
						IL-10	↑		–	–
Regulatory T cells (Treg cells)		Suppressing excessive immune responses	↓	–	(31, 74)	TGF-β	↑	(38, 165)	–	–
						IL-2	↑	(165)	–	–
						IL-10	↓	(53)	–	–
							↑	(46)	–	–
B cells		Directly participating in humoral immunity	–	↑	(6)	IL-6/STAT3	–	–	↑	(95)
Macrophages		Engulfing and digesting pathogens and secreting chemokines to recruit other immune cells	↑	↑	(15)	TNF-α	↓	(53)	–	–
							↑	(64)	–	–
						CCL-2、CCL-3、CCL-5	↑	(106)	–	–
Neutrophils		Rapid migration to the site of infection, phagocytosis and destruction of pathogens	↑	–	(31, 52, 64)	–	–	–	–	–
Natural Killer cells (NK cells)		Rapidly identifying and killing virus-infected cells	↑	–	(15, 30)	–	–	–	–	–
Complement system		Promoting pathogen clearance and activating the inflammatory response	–	–	–	C3	↑	(128)	–	–
						C3/C3a-C3aR	↑	(128)	↑	(128)
						C5a	↑	(52)	–	–
						C5/C5a-C5aR1	↑	(128)	↑	(128)
						MAC	↑	(127)	↑	(127)

Compared to healthy individuals/patients with benign breast tumors/NPM patients after treatment, “↑” means immune cells or related cytokines/molecules increasing, while “↓” means immune cells or related cytokines/molecules decreasing, and “-” means that there is a lack of research evidence in this area.

In addition, other immune-related factors (prolactin, hypersensitivity reactions due to milk stasis, lipid metabolism disorders, and immune-inflammation-related pathways) also play an important role in the pathogenesis of GLM and PCM. By summarizing the existing literature, we found that NPM is closely related to lipid metabolism, with triglyceride metabolites as the most prominent differential metabolites and arachidonic acid metabolism as the main pathway leading to lipid differences. In addition, activation of IL-6/JAK2/STAT3, PI3K/Akt/mTOR signaling pathways and NLRP3 inflammasomes may exist in PCM.

Nonetheless, studies on GLM and PCM have great research limitations. Firstly, the current studies have overlooked the examination of surface and nuclear markers of different subgroups of T cells, and only observed changes in their corresponding specific cytokines, which is relatively one-sided. Secondly, the majority of recent studies have found that GLM and PCM frequently exhibit changes in cytokines in the circulation of blood. However, GLM and PCM typically result from the local immunological inflammatory response of the breast, and cytokine changes in the serum may only be seen during the acute phase of the immune response. In addition, the small sample size of the study population and the low precision of the assay greatly limit the accuracy, confidence, and reproducibility of the results.

We must recognize that there are many research gaps in the fields of GLM and PCM. First, there is a lack of mature animal models for GLM and no recognized cellular models for either GLM or PCM, and these difficulties have limited many basic studies. Secondly, existing studies have revealed differential immune cells and cytokines in NPM (**Table 1**). Existing studies have shown that targeted inhibition of IL-6 can treat PCM in mice (97), while targeted inhibition against TNF-α alleviates the cystic neutrophil granulomatous mastitis (a type of GLM) (166, 167). However, research in this area is far from adequate and more preclinical studies targeting inhibition of elevated immune cells or cytokines in NPM are needed in the future. Finally, among other immune-related factors, the specific mechanisms of local hypersensitivity due to prolactin and milk retention in NPM remain unclear; the specific molecular mechanisms of triglyceride metabolism and arachidonic acid metabolism pathways associated with the pathogenesis of NPM and their crosstalk with the immune system remain to be elucidated; and there is a close crosstalk of the TLR with immune cells, whereas the TLR mediates multiple inflammatory pathways such as MAPK, NF-κB and PI3K/AKT in the pathogenesis of NPM need to be further explored.

In comparison to other autoimmune disorders like rheumatoid arthritis and psoriasis, research on GLM and PCM is still in its nascent stages and lacks in-depth basic research. On the one hand, we need to strengthen the research on animal and cellular models of GLM and PCM. By establishing models that are closer to the disease, we can more accurately simulate the pathogenesis of the disease and provide reliable tools for drug development and efficacy assessment. On the other hand, with the development of current detection technologies, future studies can combine single-cell sequencing technology to characterize the gene expression profiles of immune cell subpopulations present in NPM at the single-cell level, to search for subpopulations of immune cells that differ from those in the normal population, and to more accurately understand the roles of immune cells in the development of the disease and the crosstalk between immune cells. Or by combining the latest transcriptomics, proteomics, metabolomics, and other technologies, to explore the immunopathogenesis of GLM and PCM in a more comprehensive and in-depth manner. With the pathogenesis as the target, we will develop novel biomarkers and therapeutic drugs to provide new ideas and methods for the diagnosis and treatment of GLM and PCM, and bring new hope to GLM and PCM patients.

## Author contributions

YZ: Writing – original draft. JG: Writing – original draft. XD: Writing – review & editing. LS: Writing – review & editing. LL: Writing – review & editing.

## References

[1] Yuan QQ Xiao SY Farouk O Du YT Sheybani F Tan QT. MManagement of granulomatous lobular mastitis: an international multidisciplinary consensus (2021 edition). Mil Med Res. (2022) 9:20. doi: 10.1186/s40779-022-00380-5 35473758 PMC9040252

[2] Al-Khaffaf B Knox F Bundred NJ. Idiopathic granulomatous mastitis: a 25-year experience. J Am Coll Surg. (2008) 206:269–273. doi: 10.1016/j.jamcollsurg.2007.07.041 18222379

[3] Basim P Argun D Argun F. Risk factors for idiopathic granulomatous mastitis recurrence after patient-tailored treatment: do we need an escalating treatment algorithm. Breast Care (Basel). (2022) 17:172–9. doi: 10.1159/000517399 PMC9149487 35707181

[4] Tang E Ho C Chan P Chen J Goh MH Tan EY. The therapeutic dilemma of idiopathic granulomatous mastitis. Ann Acad Med Singap. (2021) 50:598–605. doi: 10.47102/annals-acadmedsg.2020645 34472554

[5] Shi L Wu J Hu Y Zhang X Li Z Xi PW. Biomedical indicators of patients with non-puerperal mastitis: A retrospective study. Nutrients. (2022) 14:4816. doi: 10.3390/nu14224816 36432503 PMC9695051

[6] Gopalakrishnan Nair C Hiran Jacob P Menon RR Misha. Inflammatory diseases of the non-lactating female breasts. Int J Surg. (2015) 13:8–11. doi: 10.1016/j.ijsu.2014.11.022 25447605

[7] Jiao Y Chang K Jiang Y Zhang J. Identification of periductal mastitis and granulomatous lobular mastitis: a literature review. Ann Transl Med. (2023) 11:158. doi: 10.21037/atm-22-6473 36846004 PMC9951018

[8] Passaro ME Broughan TA Sebek BA Esselstyn CB Jr. Lactiferous fistula. J Am Coll Surg. (1994) 178:29–32. 8156113

[9] Xing M Zhang S Zha X Zhang J. Current understanding and management of plasma cell mastitis: can we benefit from what we know. Breast Care (Basel). (2022) 17:321–9. doi: 10.1159/000517572 PMC9247483 35949416

[10] Steuer AB Stern MJ Cobos G Castilla C Joseph KA Pomeranz MK. Clinical characteristics and medical management of idiopathic granulomatous mastitis. JAMA Dermatol. (2020) 156:460–4. doi: 10.1001/jamadermatol.2019.4516 PMC6990845 31968055

[11] Barreto DS Sedgwick EL Nagi CS Benveniste AP. Granulomatous mastitis: etiology, imaging, pathology, treatment, and clinical findings. Breast Cancer Res Treat. (2018) 171:527–34. doi: 10.1007/s10549-018-4870-3 29971624

[12] Bonilla FA Oettgen HC. Adaptive immunity. J Allergy Clin Immunol. (2010) 125:S33–40. doi: 10.1016/j.jaci.2009.09.017 20061006

[13] Yigitbasi MR Guntas G Atak T Sonmez C Yalman H Uzun H. The role of interleukin-33 as an inflammatory marker in differential diagnosis of idiopathic granulomatous mastitis and breast cancer. J Invest Surg. (2017) 30:272–6. doi: 10.1080/08941939.2016.1240270 27780363

[14] Deng JQ Yu L Yang Y Feng XJ Sun J Liu J. Steroids administered after vacuum-assisted biopsy in the management of idiopathic granulomatous mastitis. J Clin Pathol. (2017) 70:827–31. doi: 10.1136/jclinpath-2016-204287 28931582

[15] Kong C Zhang C Wu Y Zeng Z Yu H Zeng J. The expression and meaning of CD68, CD163, CD57, and IgG4 in granulomatous lobular mastitis. Gland Surg. (2020) 9:936–49. doi: 10.21037/gs-20-419 PMC7475371 32953603

[16] Jiang L Li X Sun B Ma T Kong X Yang Q. Clinicopathological features of granulomatous lobular mastitis and mammary duct ectasia. Oncol Lett. (2020) 19:840–8. doi: 10.3892/ol.2019.11156 PMC6924204 31885718

[17] Li J McGregor HP. Idiopathic granulomatous mastitis associated with hyperprolactinemia: A nonoperative approach. Breast J. (2017) 23:742–4. doi: 10.1111/tbj.12914 28845595

[18] Nikolaev A Blake CN Carlson DL. Association between hyperprolactinemia and granulomatous mastitis. Breast J. (2016) 22:224–31. doi: 10.1111/tbj.12552 26705962

[19] Ruffell B Au A Rugo HS Esserman LJ Hwang ES Coussens LM. Leukocyte composition of human breast cancer. Proc Natl Acad Sci U S A. (2012) 109:2796–801. doi: 10.1073/pnas.1104303108 PMC3287000 21825174

[20] Pal B Chen Y Vaillant F Capaldo BD Joyce R Song X. A single-cell RNA expression atlas of normal, preneoplastic and tumorigenic states in the human breast. EMBO J. (2021) 40:e107333. doi: 10.15252/embj.2020107333 33950524 PMC8167363

[21] Pal B Chen Y Vaillant F Capaldo BD Joyce R Song X. A single-cell RNA expression atlas of normal, preneoplastic and tumorigenic states in the human breast. EMBO J. (2021) 40:e107333. doi: 10.15252/embj.2020107333 33950524 PMC8167363

[22] Degnim AC Brahmbhatt RD Radisky DC Hoskin TL Stallings-Mann M Laudenschlager M. Immune cell quantitation in normal breast tissue lobules with and without lobulitis. Breast Cancer Res Treat. (2014) 144:539–49. doi: 10.1007/s10549-014-2896-8 PMC3962744 24596048

[23] Wang X Han Y Liu J Zhang Y Cheng K Guo J. Exosomes play an important role in the progression of plasma cell mastitis via the PI3K-Akt-mTOR signaling pathway. Mediators Inflamm. (2019) 2019:4312016. doi: 10.1155/2019/4312016 31281227 PMC6590603

[24] Chou CH Lai SL Chen CN Lee PH Peng FC Kuo ML. IL-6 regulates Mcl-1L expression through the JAK/PI3K/Akt/CREB signaling pathway in hepatocytes: implication of an anti-apoptotic role during liver regeneration. PLoS One. (2013) 8:e66268. doi: 10.1371/journal.pone.0066268 23825534 PMC3692501

[25] Stockinger B Kassiotis G Bourgeois C. CD4 T-cell memory. Semin Immunol. (2004) 16:295–303. doi: 10.1016/j.smim.2004.08.010 15528074

[26] Roche PA Furuta K. The ins and outs of MHC class II-mediated antigen processing and presentation. Nat Rev Immunol. (2015) 15:203–16. doi: 10.1038/nri3818 PMC6314495 25720354

[27] Szabo SJ Sullivan BM Peng SL Glimcher LH. Molecular mechanisms regulating Th1 immune responses. Annu Rev Immunol. (2003) 21:713–58. doi: 10.1146/annurev.immunol.21.120601.140942 12500979

[28] Dong C. Cytokine regulation and function in T cells. Annu Rev Immunol. (2021) 39:51–76. doi: 10.1146/annurev-immunol-061020-053702 33428453

[29] Saravia J Chapman NM Chi H. Helper T cell differentiation. Cell Mol Immunol. (2019) 16:634–43. doi: 10.1038/s41423-019-0220-6 PMC6804569 30867582

[30] Emsen A Köksal H Uçaryılmaz H Kadoglou N Artaç H. The alteration of lymphocyte subsets in idiopathic granulomatous mastitis. Turk J Med Sci. (2021) 51:1905–11. doi: 10.3906/sag-2012-192 PMC8569769 33862673

[31] Dogan S Dal F Guler M Sevik H Oguz Idiz U. Is peripheral blood immunophenotyping useful to understand the etiology of Idiopathic Granulomatous. Hum Immunol. (2023) 84:315–9. doi: 10.1016/j.humimm.2023.05.001 37202243

[32] Zheng B Song J Lu M Chen C Sun S. Current research describing the role of CD4(+) T lymphocyte subsets in the pathogenesis of granulomatous lobular mastitis. J Invest Surg. (2022) 35:1790–5. doi: 10.1080/08941939.2022.2090035 36075587

[33] Sadhu S Mitra DK. Emerging concepts of adaptive immunity in leprosy. Front Immunol. (2018) 9:604. doi: 10.3389/fimmu.2018.00604 29686668 PMC5900054

[34] Basu A Ramamoorthi G Albert G Gallen C Beyer A Snyder C. Differentiation and regulation of T(H) cells: A balancing act for cancer immunotherapy. Front Immunol. (2021) 12:669474. doi: 10.3389/fimmu.2021.669474 34012451 PMC8126720

[35] Zhou L Chong MM Littman DR. Plasticity of CD4+ T cell lineage differentiation. Immunity. (2009) 30:646–55. doi: 10.1016/j.immuni.2009.05.001 19464987

[36] Hattori K Nishikawa M Watcharanurak K Ikoma A Kabashima K Toyota H. Sustained exogenous expression of therapeutic levels of IFN-gamma ameliorates atopic dermatitis in NC/Nga mice via Th1 polarization. J Immunol. (2010) 184:2729–35. doi: 10.4049/jimmunol.0900215 20107184

[37] Afkarian M Sedy JR Yang J Jacobson NG Cereb N Yang SY. T-bet is a STAT1-induced regulator of IL-12R expression in naïve CD4+ T cells. Nat Immunol. (2002) 3:549–57. doi: 10.1038/ni794 12006974

[38] Cabioglu N Cetin Aktas E Emiroglu S Tukenmez M Ozkurt E Muslumanoglu M. Ozone therapy restores immune dysfunction in refractory idiopathic granulomatous mastitis as a novel potential therapeutic approach. Cell Biol Int. (2023) 47:228–37. doi: 10.1002/cbin.11953 36378588

[39] Liu L Zhou F Wang P Yu L Ma Z Li Y. Periductal mastitis: an inflammatory disease related to bacterial infection and consequent immune responses. Mediators Inflamm. (2017) 2017:5309081. doi: 10.1155/2017/5309081 28182101 PMC5274658

[40] Liu C Yu H Chen G Yang Q Wang Z Niu N. An herbal drug combination identified by knowledge graph alleviates the clinical symptoms of plasma cell mastitis patients: A nonrandomized controlled trial. Elife. (2023) 12:e84414. doi: 10.7554/eLife.84414 36917037 PMC10063228

[41] Roan F Obata-Ninomiya K Ziegler SF. Epithelial cell-derived cytokines: more than just signaling the alarm. J Clin Invest. (2019) 129:1441–51. doi: 10.1172/JCI124606 PMC6436879 30932910

[42] Zhu J Guo L Watson CJ Hu-Li J Paul WE. Stat6 is necessary and sufficient for IL-4's role in Th2 differentiation and cell expansion. J Immunol. (2001) 166:7276–81. doi: 10.4049/jimmunol.166.12.7276 11390477

[43] Ansel KM Djuretic I Tanasa B Rao A. Regulation of Th2 differentiation and Il4 locus accessibility. Annu Rev Immunol. (2006) 24:607–56. doi: 10.1146/annurev.immunol.23.021704.115821 16551261

[44] Iwaszko M Biały S Bogunia-Kubik K. Significance of interleukin (IL)-4 and IL-13 in inflammatory arthritis. Cells. (2021) 10:3000. doi: 10.3390/cells10113000 34831223 PMC8616130

[45] Murphy E Shibuya K Hosken N Openshaw P Maino V Davis K. Reversibility of T helper 1 and 2 populations is lost after long-term stimulation. J Exp Med. (1996) 183:901–13. doi: 10.1084/jem.183.3.901 PMC2192360 8642294

[46] Koksal H Vatansev H Artac H Kadoglou N. The clinical value of interleukins-8, -10, and -17 in idiopathic granulomatous mastitis. Clin Rheumatol. (2020) 39:1671–7. doi: 10.1007/s10067-020-04925-8 31916110

[47] Arend WP Palmer G Gabay C. IL-1, IL-18, and IL-33 families of cytokines. Immunol Rev. (2008) 223:20–38. doi: 10.1111/j.1600-065X.2008.00624.x 18613828

[48] Ali S Huber M Kollewe C Bischoff SC Falk W Martin MU. IL-1 receptor accessory protein is essential for IL-33-induced activation of T lymphocytes and mast cells. Proc Natl Acad Sci U S A. (2007) 104:18660–5. doi: 10.1073/pnas.0705939104 PMC2141833 18003919

[49] Shakerian L Kolahdooz H Garousi M Keyvani V Kamal Kheder R Abdulsattar Faraj T. IL-33/ST2 axis in autoimmune disease. Cytokine. (2022) 158:156015. doi: 10.1016/j.cyto.2022.156015 36041312

[50] Zhou Y Xu Z Liu Z. Role of IL-33-ST2 pathway in regulating inflammation: current evidence and future perspectives. J Transl Med. (2023) 21:902. doi: 10.1186/s12967-023-04782-4 38082335 PMC10714644

[51] Haghbin M Sotoodeh Jahromi A Ranjbaran R Abbasi M Hashemi Tayer A. Comparison of interleukin-33 serum levels in patients with breast cancer and idiopathic granulomatous mastitis. Asian Pac J Cancer Prev. (2023) 24:1629–34. doi: 10.31557/APJCP.2023.24.5.1629 PMC10495896 37247282

[52] Esmaeil NK Salih AM Hammood ZD Pshtiwan L Abdullah AM Kakamad FH. Clinical, microbiological, immunological and hormonal profiles of patients with granulomatous mastitis. BioMed Rep. (2023) 18:41. doi: 10.3892/br.2023.1624 37325183 PMC10265128

[53] Cakir C Nayci AE Ferlengez E Guler M Idiz UO. Cytokines the etiology of idiopathic granulomatous mastitis. J Coll Physicians Surg Pak. (2022) 32:869–73. doi: 10.29271/jcpsp.2022.07.869 35795934

[54] Lee GR. The balance of Th17 versus Treg cells in autoimmunity. Int J Mol Sci. (2018) 19:730. doi: 10.3390/ijms19030730 29510522 PMC5877591

[55] Bettelli E Carrier Y Gao W Korn T Strom TB Oukka M. Reciprocal developmental pathways for the generation of pathogenic effector TH17 and regulatory T cells. Nature. (2006) 441:235–8. doi: 10.1038/nature04753 16648838

[56] Korn T Bettelli E Oukka M Kuchroo VK. IL-17 and Th17 cells. Annu Rev Immunol. (2009) 27:485–517. doi: 10.1146/annurev.immunol.021908.132710 19132915

[57] Huangfu L Li R Huang Y Wang S. The IL-17 family in diseases: from bench to bedside. Signal Transduct Target Ther. (2023) 8:402. doi: 10.1038/s41392-023-01620-3 37816755 PMC10564932

[58] Maddur MS Miossec P Kaveri SV Bayry J. Th17 cells: biology, pathogenesis of autoimmune and inflammatory diseases, and therapeutic strategies. Am J Pathol. (2012) 181:8–18. doi: 10.1016/j.ajpath.2012.03.044 22640807

[59] Wu B Wan Y. Molecular control of pathogenic Th17 cells in autoimmune diseases. Int Immunopharmacol. (2020) 80:106187. doi: 10.1016/j.intimp.2020.106187 31931372 PMC7031035

[60] Lowes MA Kikuchi T Fuentes-Duculan J Cardinale I Zaba LC Haider AS. Psoriasis vulgaris lesions contain discrete populations of Th1 and Th17 T cells. J Invest Dermatol. (2008) 128:1207–11. doi: 10.1038/sj.jid.5701213 18200064

[61] Gu C Wu L Li X. IL-17 family: cytokines, receptors and signaling. Cytokine. (2013) 64:477–85. doi: 10.1016/j.cyto.2013.07.022 PMC3867811 24011563

[62] Pandolfi F Franza L Carusi V Altamura S Andriollo G Nucera E. Interleukin-6 in rheumatoid arthritis. Int J Mol Sci. (2020) 21:5238. doi: 10.3390/ijms21155238 32718086 PMC7432115

[63] Tanaka T Narazaki M Kishimoto T. IL-6 in inflammation, immunity, and disease. Cold Spring Harb Perspect Biol. (2014) 6:a016295. doi: 10.1101/cshperspect.a016295 25190079 PMC4176007

[64] Zhou Y Wu J Ma L Wang B Meng T Chen H. Differences and significance of peripheral blood interleukin-6 expression between patients with granulomatous lobular mastitis and those with benign breast tumors. Front Med (Lausanne). (2023) 10:1273406. doi: 10.3389/fmed.2023.1273406 37817809 PMC10561106

[65] Huang YM Lo C Cheng CF Lu CH Hsieh SC Li KJ. Serum C-reactive protein and interleukin-6 levels as biomarkers for disease severity and clinical outcomes in patients with idiopathic granulomatous mastitis. J Clin Med. (2021) 10. doi: 10.3390/jcm10102077 PMC8150275 34066203

[66] Schinocca C Rizzo C Fasano S Grasso G La Barbera L Ciccia F. Role of the IL-23/IL-17 pathway in rheumatic diseases: an overview. Front Immunol. (2021) 12:637829. doi: 10.3389/fimmu.2021.637829 33692806 PMC7937623

[67] Saydam M Yilmaz KB Sahin M Yanik H Akinci M Yilmaz I. New findings on autoimmune etiology of idiopathic granulomatous mastitis: serum IL-17, IL-22 and IL-23 levels of patients. J Invest Surg. (2021) 34:993–7. doi: 10.1080/08941939.2020.1725190 32046543

[68] Ujiie H. Regulatory T cells in autoimmune skin diseases. Exp Dermatol. (2019) 28:642–6. doi: 10.1111/exd.13535 29575350

[69] Sakaguchi S Mikami N Wing JB Tanaka A Ichiyama K Ohkura N. Regulatory T cells and human disease. Annu Rev Immunol. (2020) 38:541–66. doi: 10.1146/annurev-immunol-042718-041717 32017635

[70] Wing JB Tanaka A Sakaguchi S. Human FOXP3(+) regulatory T cell heterogeneity and function in autoimmunity and cancer. Immunity. (2019) 50:302–16. doi: 10.1016/j.immuni.2019.01.020 30784578

[71] Pawlak M Ho AW Kuchroo VK. Cytokines and transcription factors in the differentiation of CD4(+) T helper cell subsets and induction of tissue inflammation and autoimmunity. Curr Opin Immunol. (2020) 67:57–67. doi: 10.1016/j.coi.2020.09.001 33039897 PMC7747296

[72] Noack M Miossec P. Th17 and regulatory T cell balance in autoimmune and inflammatory diseases. Autoimmun Rev. (2014) 13:668–77. doi: 10.1016/j.autrev.2013.12.004 24418308

[73] Palomares O Martín-Fontecha M Lauener R Traidl-Hoffmann C Cavkaytar O Akdis M. Regulatory T cells and immune regulation of allergic diseases: roles of IL-10 and TGF-β. Genes Immun. (2014) 15:511–20. doi: 10.1038/gene.2014.45 25056447

[74] Ucaryilmaz H Koksal H Emsen A Kadoglou N Dixon JM Artac H. The role of regulatory T and B cells in the etiopathogenesis of idiopathic granulomatous mastitis. Immunol Invest. (2022) 51:357–67. doi: 10.1080/08820139.2020.1832114 33034215

[75] Jiang Q Yang G Liu Q Wang S Cui D. Function and role of regulatory T cells in rheumatoid arthritis. Front Immunol. (2021) 12:626193. doi: 10.3389/fimmu.2021.626193 33868244 PMC8047316

[76] Li Y Chen L Zhang C Wang Y Hu J Zhou M. Clinicopathologic features and pathogens of granulomatous lobular mastitis. Breast Care (Basel). (2023) 18:130–40. doi: 10.1159/000529391 PMC10228255 37261131

[77] Reina-Campos M Scharping NE Goldrath AW. CD8(+) T cell metabolism in infection and cancer. Nat Rev Immunol. (2021) 21:718–38. doi: 10.1038/s41577-021-00537-8 PMC8806153 33981085

[78] Harty JT Tvinnereim AR White DW. CD8+ T cell effector mechanisms in resistance to infection. Annu Rev Immunol. (2000) 18:275–308. doi: 10.1146/annurev.immunol.18.1.275 10837060

[79] Zhou Z He H Wang K Shi X Wang Y Su Y. Granzyme A from cytotoxic lymphocytes cleaves GSDMB to trigger pyroptosis in target cells. Science. (2020) 368:eaaz7548. doi: 10.1126/science.aaz7548 32299851

[80] Tian L Zhou W Wu X Hu Z Qiu L Zhang H. CTLs: Killers of intracellular bacteria. Front Cell Infect Microbiol. (2022) 12:967679. doi: 10.3389/fcimb.2022.967679 36389159 PMC9645434

[81] Zuo XM Wang TS Shi XG Gao X Gao S Sun P. Pyroptosis: the pathological process that dominates granulomatous lobular mastitis. J Physiol Pharmacol. (2021) 72:469–475. doi: 10.26402/jpp.2021.3.15 34873070

[82] Shalini S Dorstyn L Dawar S Kumar S. Old, new and emerging functions of caspases. Cell Death Differ. (2015) 22:526–39. doi: 10.1038/cdd.2014.216 PMC4356345 25526085

[83] Hu Q Cui X Tao L Xiu L Wang T Wang X. Staphylococcus aureus induces apoptosis in primary bovine mammary epithelial cells through Fas-FADD death receptor-linked caspase-8 signaling. DNA Cell Biol. (2014) 33:388–97. doi: 10.1089/dna.2013.2195 24564258

[84] Payandeh Z Bahrami AA Hoseinpoor R Mortazavi Y Rajabibazl M Rahimpour A. The applications of anti-CD20 antibodies to treat various B cells disorders. BioMed Pharmacother. (2019) 109:2415–26. doi: 10.1016/j.biopha.2018.11.121 30551501

[85] Rodman JS Ingleby H. PLASMA CELL MASTITIS. Ann Surg. (1939) 109:921–30. doi: 10.1097/00000658-193906000-00003 PMC1391283 17857379

[86] Dong Y Pi X Bartels-Burgahn F Saltukoglu D Liang Z Yang J. Structural principles of B cell antigen receptor assembly. Nature. (2022) 612:156–61. doi: 10.1038/s41586-022-05412-7 PMC10499536 36228656

[87] Huse K Bai B Hilden VI Bollum LK Våtsveen TK Munthe LA. Mechanism of CD79A and CD79B support for IgM+ B cell fitness through B cell receptor surface expression. J Immunol. (2022) 209:2042–53. doi: 10.4049/jimmunol.2200144 PMC9643646 36426942

[88] Tiburzy B Kulkarni U Hauser AE Abram M Manz RA. Plasma cells in immunopathology: concepts and therapeutic strategies. Semin Immunopathol. (2014) 36:277–88. doi: 10.1007/s00281-014-0426-8 24740168

[89] Tumang JR Hsia CY Tian W Bromberg JF Liou HC. IL-6 rescues the hyporesponsiveness of c-Rel deficient B cells independent of Bcl-xL, Mcl-1, and Bcl-2. Cell Immunol. (2002) 217:47–57. doi: 10.1016/s0008-8749(02)00513-0 12426000

[90] Xu J Cao K Liu X Zhao L Feng Z Liu J. Punicalagin regulates signaling pathways in inflammation-associated chronic diseases. Antioxidants (Basel). (2021) 11:29. doi: 10.3390/antiox11010029 35052533 PMC8773334

[91] Rose-John S. IL-6 trans-signaling via the soluble IL-6 receptor: importance for the pro-inflammatory activities of IL-6. Int J Biol Sci. (2012) 8:1237–47. doi: 10.7150/ijbs.4989 PMC3491447 23136552

[92] Chen X Han K Lin G Liu C Wang S Shi X. Ctenopharyngodon Idella STAT3 alleviates autophagy by up-regulating BCL-2 expression. Fish Shellfish Immunol. (2019) 91:194–201. doi: 10.1016/j.fsi.2019.05.034 31108175

[93] Kotov JA Xu Y Carey ND Cyster JG. LTβR overexpression promotes plasma cell accumulation. PLoS One. (2022) 17:e0270907. doi: 10.1371/journal.pone.0270907 35925983 PMC9352096

[94] Lernoux M Schnekenburger M Dicato M Diederich M. Susceptibility of multiple myeloma to B-cell lymphoma 2 family inhibitors. Biochem Pharmacol. (2021) 188:114526. doi: 10.1016/j.bcp.2021.114526 33741332

[95] Liu Y Zhang J Zhou YH Jiang YN Zhang W Tang XJ. IL-6/STAT3 signaling pathway is activated in plasma cell mastitis. Int J Clin Exp Pathol. (2015) 8:12541–8. PMC4680387 26722442

[96] Liu Y Zhang J Zhou YH Zhang HM Wang K Ren Y. Activation of the IL-6/JAK2/STAT3 pathway induces plasma cell mastitis in mice. Cytokine. (2018) 110:150–8. doi: 10.1016/j.cyto.2018.05.002 29751177

[97] Liu Y Sun Y Zhou Y Tang X Wang K Ren Y. Sinomenine hydrochloride inhibits the progression of plasma cell mastitis by regulating IL-6/JAK2/STAT3 pathway. Int Immunopharmacol. (2020) 81:106025. doi: 10.1016/j.intimp.2019.106025 31810886

[98] Nutt SL Hodgkin PD Tarlinton DM Corcoran LM. The generation of antibody-secreting plasma cells. Nat Rev Immunol. (2015) 15:160–71. doi: 10.1038/nri3795 25698678

[99] Cheuk W Chan AC Lam WL Chow SM Crowley P Lloydd R. IgG4-related sclerosing mastitis: description of a new member of the IgG4-related sclerosing diseases. Am J Surg Pathol. (2009) 33:1058–64. doi: 10.1097/PAS.0b013e3181998cbe 19384187

[100] Chougule A Bal A Das A Singh G. IgG4 related sclerosing mastitis: expanding the morphological spectrum of IgG4 related diseases. Pathology. (2015) 47:27–33. doi: 10.1097/PAT.0000000000000187 25474510

[101] Drent M Crouser ED Grunewald J. Challenges of sarcoidosis and its management. N Engl J Med. (2021) 385:1018–32. doi: 10.1056/NEJMra2101555 34496176

[102] Krausgruber T Redl A Barreca D Doberer K Romanovskaia D Dobnikar L. Single-cell and spatial transcriptomics reveal aberrant lymphoid developmental programs driving granuloma formation. Immunity. (2023) 56:289–306.e7. doi: 10.1016/j.immuni.2023.01.014 36750099 PMC9942876

[103] Pagán AJ Ramakrishnan L. The formation and function of granulomas. Annu Rev Immunol. (2018) 36:639–65. doi: 10.1146/annurev-immunol-032712-100022 29400999

[104] Gupta RK. Fine needle aspiration cytology of granulomatous mastitis: a study of 18 cases. Acta Cytol. (2010) 54:138–41. doi: 10.1159/000324998 20391968

[105] Peng Y Zhou M Yang H Qu R Qiu Y Hao J. Regulatory mechanism of M1/M2 macrophage polarization in the development of autoimmune diseases. Mediators Inflamm. (2023) 2023:8821610. doi: 10.1155/2023/8821610 37332618 PMC10270764

[106] Wang Z Wang N Liu X Wang Q Xu B Liu P. Broadleaf Mahonia attenuates granulomatous lobular mastitis−associated inflammation by inhibiting CCL−5 expression in macrophages. Int J Mol Med. (2018) 41:340–52. doi: 10.3892/ijmm.2017.3246 PMC5746325 29138800

[107] Ross EA Devitt A Johnson JR. Macrophages: the good, the bad, and the gluttony. Front Immunol. (2021) 12:708186. doi: 10.3389/fimmu.2021.708186 34456917 PMC8397413

[108] Pauleau AL Murray PJ. Role of nod2 in the response of macrophages to toll-like receptor agonists. Mol Cell Biol. (2003) 23:7531–9. doi: 10.1128/MCB.23.21.7531-7539.2003 PMC207570 14560001

[109] Watanabe T Kitani A Murray PJ Strober W. NOD2 is a negative regulator of Toll-like receptor 2-mediated T helper type 1 responses. Nat Immunol. (2004) 5:800–8. doi: 10.1038/ni1092 15220916

[110] Bercot B Kannengiesser C Oudin C Grandchamp B Sanson-le Pors MJ Mouly S. First description of NOD2 variant associated with defective neutrophil responses in a woman with granulomatous mastitis related to corynebacteria. J Clin Microbiol. (2009) 47:3034–7. doi: 10.1128/JCM.00561-09 PMC2738115 19641059

[111] Liew PX Kubes P. The neutrophil's role during health and disease. Physiol Rev. (2019) 99:1223–48. doi: 10.1152/physrev.00012.2018 30758246

[112] Zhang H Wang Y Qu M Li W Wu D Cata JP. Neutrophil, neutrophil extracellular traps and endothelial cell dysfunction in sepsis. Clin Transl Med. (2023) 13:e1170. doi: 10.1002/ctm2.1170 36629024 PMC9832433

[113] Gong T Liu L Jiang W Zhou R. DAMP-sensing receptors in sterile inflammation and inflammatory diseases. Nat Rev Immunol. (2020) 20:95–112. doi: 10.1038/s41577-019-0215-7 31558839

[114] Watts ER Walmsley SR. Getting DAMP(s) wets the whistle for neutrophil recruitment. Immunity. (2018) 48:846–8. doi: 10.1016/j.immuni.2018.04.027 29768171

[115] Li Q Wan J Feng Z Shi J Wei W. Predictive significance of the preoperative neutrophil-lymphocyte ratio for recurrence in idiopathic granulomatous mastitis patients. Am Surg. (2023) 89:5577–83. doi: 10.1177/00031348231161793 36880848

[116] Ravindran M Khan MA Palaniyar N. Neutrophil extracellular trap formation: physiology, pathology, and pharmacology. Biomolecules. (2019) 9:365. doi: 10.3390/biom9080365 31416173 PMC6722781

[117] Hidalgo A Libby P Soehnlein O Aramburu IV Papayannopoulos V Silvestre-Roig C. Neutrophil extracellular traps: from physiology to pathology. Cardiovasc Res. (2022) 118:2737–53. doi: 10.1093/cvr/cvab329 PMC9586562 34648022

[118] Papayannopoulos V. Neutrophil extracellular traps in immunity and disease. Nat Rev Immunol. (2018) 18:134–47. doi: 10.1038/nri.2017.105 28990587

[119] Liu M Liang S Zhang C. NK cells in autoimmune diseases: protective or pathogenic. Front Immunol. (2021) 12:624687. doi: 10.3389/fimmu.2021.624687 33777006 PMC7994264

[120] Vivier E Raulet DH Moretta A Caligiuri MA Zitvogel L Lanier LL. Innate or adaptive immunity? The example of natural killer cells. Science. (2011) 331:44–9. doi: 10.1126/science.1198687 PMC3089969 21212348

[121] Schoenborn JR Wilson CB. Regulation of interferon-gamma during innate and adaptive immune responses. Adv Immunol. (2007) 96:41–101. doi: 10.1016/S0065-2776(07)96002-2 17981204

[122] Halbgebauer R Schmidt CQ Karsten CM Ignatius A Huber-Lang M. Janus face of complement-driven neutrophil activation during sepsis. Semin Immunol. (2018) 37:12–20. doi: 10.1016/j.smim.2018.02.004 29454576

[123] Bjanes E Nizet V. More than a pore: nonlytic antimicrobial functions of complement and bacterial strategies for evasion. Microbiol Mol Biol Rev. (2021) 85:e00177–20. doi: 10.1128/MMBR.00177-20 PMC8549852 33504655

[124] Lubbers R van Essen MF van Kooten C Trouw LA. Production of complement components by cells of the immune system. Clin Exp Immunol. (2017) 188:183–94. doi: 10.1111/cei.12952 PMC5383442 28249350

[125] Fishelson Z Kirschfink M. Complement C5b-9 and cancer: mechanisms of cell damage, cancer counteractions, and approaches for intervention. Front Immunol. (2019) 10:752. doi: 10.3389/fimmu.2019.00752 31024572 PMC6467965

[126] Cravedi P Leventhal J Lakhani P Ward SC Donovan MJ Heeger PS. Immune cell-derived C3a and C5a costimulate human T cell alloimmunity. Am J Transpl. (2013) 13:2530–9. doi: 10.1111/ajt.12405 PMC3809075 24033923

[127] Zhang HJ Ding PP Zhang XS Wang XC Sun DW Bu QA. MAC mediates mammary duct epithelial cell injury in plasma cell mastitis and granulomatous mastitis. Int Immunopharmacol. (2022) 113:109303. doi: 10.1016/j.intimp.2022.109303 36252469

[128] Li XQ Sun HG Wang XH Zhang HJ Zhang XS Yu Y. Activation of C3 and C5 may be involved in the inflammatory progression of PCM and GM. Inflammation. (2022) 45:739–52. doi: 10.1007/s10753-021-01580-2 34997873

[129] Bui TM Wiesolek HL Sumagin R. ICAM-1: A master regulator of cellular responses in inflammation, injury resolution, and tumorigenesis. J Leukoc Biol. (2020) 108:787–99. doi: 10.1002/JLB.2MR0220-549R PMC7977775 32182390

[130] Homeister JW Zhang M Frenette PS Hynes RO Wagner DD Lowe JB. Overlapping functions of E- and P-selectin in neutrophil recruitment during acute inflammation. Blood. (1998) 92:2345–52. doi: 10.1182/blood.V92.7.2345 9746773

[131] Dong Y Yu JJ Shibahara Y Lu HS He HY Liu JD. Intercellular adhesion molecule 1/2 and E-selectin in plasma cell mastitis: immunohistochemical study of 35 cases. Hum Pathol. (2014) 45:606–10. doi: 10.1016/j.humpath.2013.06.025 24457076

[132] Pluguez-Turull CW Nanyes JE Quintero CJ Alizai H Mais DD Kist KA. Idiopathic granulomatous mastitis: manifestations at multimodality imaging and pitfalls. Radiographics. (2018) 38:330–56. doi: 10.1148/rg.2018170095 29528819

[133] Bi J Li Z Lin X Li F Xu H Yu X. Etiology of granulomatous lobular mastitis based on metagenomic next-generation sequencing. Int J Infect Dis. (2021) 113:243–50. doi: 10.1016/j.ijid.2021.10.019 34673215

[134] Borba VV Zandman-Goddard G Shoenfeld Y. Prolactin and autoimmunity. Front Immunol. (2018) 9:73. doi: 10.3389/fimmu.2018.00073 29483903 PMC5816039

[135] Vera-Lastra O Jara LJ Espinoza LR. Prolactin and autoimmunity. Autoimmun Rev. (2002) 1:360–4. doi: 10.1016/s1568-9972(02)00081-2 12848992

[136] Brand JM Frohn C Cziupka K Brockmann C Kirchner H Luhm J. Prolactin triggers pro-inflammatory immune responses in peripheral immune cells. Eur Cytokine Netw. (2004) 15:99–104. 15319167

[137] Tomio A Schust DJ Kawana K Yasugi T Kawana Y Mahalingaiah S. Prolactin can modulate CD4+ T-cell response through receptor-mediated alterations in the expression of T-bet. Immunol Cell Biol. (2008) 86:616–21. doi: 10.1038/icb.2008.29 18414429

[138] Kalsi AK Halder A Jain M Chaturvedi PK Mathew M Sharma JB. Association of raised levels of IL-4 and anti-TPO with hyperprolactinemia. Am J Reprod Immunol. (2019) 81:e13085. doi: 10.1111/aji.13085 30614113

[139] Krause I Blumenfeld Z Malchinsky M Cohen S Blank M Eldor A. Anti-endothelial cell antibodies in the sera of hyperprolactinemic women. Lupus. (1998) 7:377–82. doi: 10.1191/096120398678920316 9736319

[140] Sun J Shao S Wan H Wu X Feng J Gao Q. Prediction models for postoperative recurrence of non-lactating mastitis based on machine learning. BMC Med Inform Decis Mak. (2024) 24:106. doi: 10.1186/s12911-024-02499-y 38649879 PMC11036744

[141] Ge T Sun P Feng X Gao X Gao S Wang T. Clinical features and risk factors of bilateral granulomatous lobular mastitis. Med (Baltimore). (2024) 103:e37854. doi: 10.1097/MD.0000000000037854 PMC11049733 38669433

[142] Quarrie LH Addey CV Wilde CJ. Programmed cell death during mammary tissue involution induced by weaning, litter removal, and milk stasis. J Cell Physiol. (1996) 168:559–69. doi: 10.1002/(SICI)1097-4652(199609)168:3&lt;559::AID-JCP8<3.0.CO;2-O 8816910

[143] Humphreys RC Bierie B Zhao L Raz R Levy D Hennighausen L. Deletion of Stat3 blocks mammary gland involution and extends functional competence of the secretory epithelium in the absence of lactogenic stimuli. Endocrinology. (2002) 143:3641–50. doi: 10.1210/en.2002-220224 12193580

[144] Jeong J Lee J Talaia G Kim W Song J Hong J. Intracellular calcium links milk stasis to lysosome-dependent cell death during early mammary gland involution. Cell Mol Life Sci. (2024) 81:29. doi: 10.1007/s00018-023-05044-8 38212474 PMC10784359

[145] Wöckel A Abou-Dakn M Beggel A Arck P. Inflammatory breast diseases during lactation: health effects on the newborn-a literature review. Mediators Inflamm. (2008) 2008:298760. doi: 10.1155/2008/298760 18437232 PMC2324165

[146] Nickerson SC. Immunological aspects of mammary involution. J Dairy Sci. (1989) 72:1665–78. doi: 10.3168/jds.S0022-0302(89)79278-X 2668362

[147] Andersen CJ. Lipid metabolism in inflammation and immune function. Nutrients. (2022) 14:1414. doi: 10.3390/nu14071414 35406026 PMC9002396

[148] Fernandes das Neves M Batuca JR Delgado Alves J. The role of high-density lipoprotein in the regulation of the immune response: implications for atherosclerosis and autoimmunity. Immunology. (2021) 164:231–41. doi: 10.1111/imm.13348 PMC8442240 33934336

[149] Morris G Gevezova M Sarafian V Maes M. Redox regulation of the immune response. Cell Mol Immunol. (2022) 19:1079–101. doi: 10.1038/s41423-022-00902-0 PMC9508259 36056148

[150] Chen X Shao S Wu X Feng J Qu W Gao Q. LC/MS-based untargeted lipidomics reveals lipid signatures of nonpuerperal mastitis. Lipids Health Dis. (2023) 22:122. doi: 10.1186/s12944-023-01887-z 37553678 PMC10408177

[151] Zhang Y Liu Y Sun J Zhang W Guo Z Ma Q. Arachidonic acid metabolism in health and disease. MedComm (2020). (2023) 4:e363. doi: 10.1002/mco2.363 37746665 PMC10511835

[152] Kawai T Akira S. Toll-like receptors and their crosstalk with other innate receptors in infection and immunity. Immunity. (2011) 34:637–50. doi: 10.1016/j.immuni.2011.05.006 21616434

[153] Kumar V. Toll-like receptors in adaptive immunity. Handb Exp Pharmacol. (2022) 276:95–131. doi: 10.1007/164_2021_543 34510306

[154] Mandraju R Murray S Forman J Pasare C. Differential ability of surface and endosomal TLRs to induce CD8 T cell responses. vivo J Immunol. (2014) 192:4303–15. doi: 10.4049/jimmunol.1302244 PMC4002505 24688022

[155] Karim AF Reba SM Li Q Boom WH Rojas RE. Toll like Receptor 2 engagement on CD4(+) T cells promotes TH9 differentiation and function. Eur J Immunol. (2017) 47:1513–24. doi: 10.1002/eji.201646846 PMC5606324 28665005

[156] Peng G Guo Z Kiniwa Y Voo KS Peng W Fu T. Toll-like receptor 8-mediated reversal of CD4+ regulatory T cell function. Science. (2005) 309:1380–4. doi: 10.1126/science.1113401 16123302

[157] McGuire VA Arthur JS. Subverting toll-like receptor signaling by bacterial pathogens. Front Immunol. (2015) 6:607. doi: 10.3389/fimmu.2015.00607 26648936 PMC4664646

[158] Troutman TD Bazan JF Pasare C. Toll-like receptors, signaling adapters and regulation of the pro-inflammatory response by PI3K. Cell Cycle. (2012) 11:3559–67. doi: 10.4161/cc.21572 PMC3478307 22895011

[159] Hawkins PT Stephens LR. PI3K signalling in inflammation. Biochim Biophys Acta. (2015) 1851:882–97. doi: 10.1016/j.bbalip.2014.12.006 25514767

[160] Strauss L Czystowska M Szajnik M Mandapathil M Whiteside TL. Differential responses of human regulatory T cells (Treg) and effector T cells to rapamycin. PLoS One. (2009) 4:e5994. doi: 10.1371/journal.pone.0005994 19543393 PMC2694984

[161] Yang K Chi H. mTOR and metabolic pathways in T cell quiescence and functional activation. Semin Immunol. (2012) 24:421–8. doi: 10.1016/j.smim.2012.12.004 PMC3855395 23375549

[162] Zhang W Li G Luo R Lei J Song Y Wang B. Cytosolic escape of mitochondrial DNA triggers cGAS-STING-NLRP3 axis-dependent nucleus pulposus cell pyroptosis. Exp Mol Med. (2022) 54:129–42. doi: 10.1038/s12276-022-00729-9 PMC8894389 35145201

[163] Chen Y Ye X Escames G Lei W Zhang X Li M. The NLRP3 inflammasome: contributions to inflammation-related diseases. Cell Mol Biol Lett. (2023) 28:51. doi: 10.1186/s11658-023-00462-9 37370025 PMC10303833

[164] Sun X Hou J Ni T Xu Z Yan W Kong L. MCC950 attenuates plasma cell mastitis in an MDSC-dependent manner. Int Immunopharmacol. (2024) 131:111803. doi: 10.1016/j.intimp.2024.111803 38460298

[165] Chen J Yang J Qin Y Sun C Xu J Zhou X. Tongue features of patients with granulomatous lobular mastitis. Med (Baltimore). (2022) 101:e31327. doi: 10.1097/MD.0000000000031327 PMC9678557 36401439

[166] Chiu LW Goodwin K Vohra P Amerson E. Cystic neutrophilic granulomatous mastitis regression with the tumor necrosis factor-α Inhibitor, adalimumab. Eur J Breast Health. (2022) 18:94–101. doi: 10.4274/ejbh.galenos.2021.2021-7-2 35059598 PMC8734519

[167] Kamat S Schaffenburg W Bongiorno M. Cystic neutrophilic granulomatous mastitis treatment with consecutive dapsone and adalimumab. Dermatopathology (Basel). (2022) 9:408–12. doi: 10.3390/dermatopathology9040047 PMC9777493 36547221

